# The GPCR adaptor protein Norbin controls the trafficking of C5aR1 and CXCR4 in mouse neutrophils

**DOI:** 10.1016/j.jbc.2024.107940

**Published:** 2024-10-28

**Authors:** Stephen A. Chetwynd, Richard J. Ward, Graeme Milligan, Heidi C.E. Welch

**Affiliations:** 1 Signalling Programme, The Babraham Institute, Babraham Research Campus, Cambridge, UK; 2 Centre for Translational Pharmacology, School of Molecular Biosciences, College of Medical, Veterinary and Life Sciences, University of Glasgow, Glasgow, UK

**Keywords:** agonist-induced internalization, β-arrestin, C5aR1, CXCR4, G protein–coupled receptor (GPCR), GPCR trafficking, Ncdn, Neurochondrin, P-Rex1, receptor desensitization, receptor endocytosis, receptor recycling

## Abstract

Norbin (Neurochondrin, NCDN) is a G protein–coupled receptor (GPCR) adaptor protein known for its importance in neuronal function. Norbin works by binding to numerous GPCRs, controlling their steady-state trafficking and sometimes their agonist-induced internalization, as well as their signaling. We recently showed that Norbin is expressed in neutrophils, limits the surface levels of the GPCRs C5aR1 and CXCR4 in neutrophils, and suppresses neutrophil-mediated innate immunity. Here, we identify C5aR1 and CXCR4 as direct Norbin interactors and used mice with myeloid-Norbin deficiency to investigate the role of Norbin in the trafficking of endogenous C5aR1 and CXCR4 in primary neutrophils by flow cytometry and cell fractionation. We show that Norbin mediates the agonist-induced internalization of C5aR1 through a β-arrestin–dependent mechanism and limits the recycling of internalized C5aR1 and CXCR4 back to the cell surface. Norbin does not control the constitutive internalization of C5aR1 and CXCR4 nor does it affect the agonist-induced internalization of CXCR4. Norbin suppresses C5aR1 signaling in mouse neutrophils by limiting the C5a-stimulated membrane translocation of Tiam1, Vav, and PKCδ, and activation of Erk and p38 Mapk pathways, as well as Gα_i_-dependent reactive oxygen species production. Our study demonstrates how Norbin suppresses C5aR1 and CXCR4 function in neutrophils and increases our understanding of the mechanisms through which Norbin regulates GPCR trafficking generally, by identifying its importance in β-arrestin recruitment, β-arrestin dependent agonist-induced receptor internalization, and receptor recycling.

Norbin (Neurochondrin, NCDN) is an essential, highly conserved 79 kDa cytosolic adapter protein with no catalytic activity, homology to other proteins, or protein domains (1). For many years, Norbin was almost exclusively studied in the context of neurons where it is highly expressed and plays important regulatory roles in neuronal morphology and synaptic plasticity (2, 3, 4, 5, 6, 7, 8). General Norbin deficiency in mice is early embryonic lethal (9), and conditional deficiencies in parts of the nervous system result in spatial learning defects, epileptic seizures, impaired cognitive functions, and depression- or schizophrenia-like behaviors (9, 10, 11). More recently, Norbin was shown to be expressed in a range of other cell types, including neutrophils, and we identified the protein to function as a suppressor of neutrophil-mediated innate immunity and of neutrophil effector responses, including the production of reactive oxygen species (ROS) and neutrophil extracellular traps (5, 12).

The mechanisms through which Norbin exerts its biological roles are still poorly understood. Norbin interacts with a range of proteins and lipids, including the Rac-GEF P-Rex1 (5), the Rho-GTPase effector Dia1 (4), the PKA regulatory subunit RIIα (13), the plexin receptor Semaphorin 4C (14), and the lipid second messenger phosphatidic acid (1, 15). However, the best-defined mechanism of Norbin function is its direct binding to the intracellular C-terminal tails of numerous G protein–coupled receptors (GPCRs), which enables it to regulate GPCR signaling and trafficking. Norbin was shown to interact directly with 35 out of 55 GPCRs tested to date (1). Commonly, Norbin binding leads to the upregulation or downregulation of the steady-state cell surface levels of its target GPCRs, in a context-dependent manner. For example, the surface level of the metabotropic glutamate receptor mGluR_5_ was elevated upon Norbin expression in neuroblastic Neuro2a cells (8), and knockdown of Norbin in primary rodent neurons reduced mGluR_1_ and mGluR_5_ surface levels (16). Similarly, the surface level of melanin-concentrating hormone receptor 1 (MCH1) in HEK293 cells was increased upon Norbin expression (17). In contrast, the surface level of sphingosine 1-phosphate receptor 1 (S1PR1), another direct Norbin target (18), was elevated in Norbin-deficient PC12 pheochromocytoma cells (19). Unlike surface levels, the total cellular levels of mGluR_5_, MCH1, and S1PR1 were unaffected by Norbin expression (8, 18, 19). In addition to steady-state GPCR trafficking, Norbin can also control the agonist-induced internalization of some, but not all, its target GPCRs. Norbin expression promotes the agonist-dependent internalization of mGluR_1_ and mGluR_5_ in rodent cortical and hippocampal neurons (16), and of S1PR1 in PC12 cells (19), but not that of MCH1 in HEK293 cells (17). Norbin affects GPCR signaling as well as trafficking, notably the Ca^2+^ and Erk pathways (1), and use of GPCR mutants deficient in Norbin binding showed that the direct interaction between GPCRs and Norbin is critical for its effects on GPCR trafficking and signaling (8).

We recently reported elevated surface levels of the GPCRs C5aR1 and CXCR4 in neutrophils from mice with myeloid-Norbin deficiency (*Ncdn*^*Δmye*^) (12). C5aR1 is the receptor for complement component 5a (C5a), an anaphylatoxin with potent proinflammatory and chemotactic properties which is rapidly produced by the complement system during the early acute stages of inflammation and infection (20, 21, 22, 23). C5aR1 is widely expressed, but particularly in myeloid cells such as neutrophils and macrophages, and couples predominately to Gα_i_ (24, 25). C5aR1 is found at low levels on the surface of basal neutrophils, as it is largely stored on the membrane of neutrophil granules and becomes upregulated to the cell surface by degranulation (12, 26). Upon activation by C5a, the C-terminal tail of C5aR1 is phosphorylated at multiple serine and threonine residues by G protein–coupled receptor kinase (GRK) and PKC (27, 28). Phosphorylation of S334 and S338 is critical for the C5a-induced stable recruitment of β-arrestin and internalization of C5aR1, and for the attenuation of C5a-induced calcium mobilization, Erk2 activity, and ROS production in promyeloblastic HL60 cells (29, 30, 31). CXCR4 is the receptor for stromal cell–derived factor 1α (SDF1α), a ligand that is continually produced by bone marrow stromal cells. CXCR4 plays important roles in regulating the bone marrow retention and clearance of neutrophils (32). Downregulation of SDF1α production in bone marrow during the early stages of acute inflammation increases the release of neutrophils into the circulation (33). Like C5aR1, CXCR4 predominantly couples to Gα_i_ proteins, and its activation regulates cell polarity and migration (34). Upon SDF1α stimulation, CXCR4 is rapidly phosphorylated at multiple serine and threonine residues in its C-terminal tail (35). S346/S347 phosphorylation, mediated by GRK2/3, precedes PKC-dependent phosphorylation of S324/325, and GRK6-mediated phosphorylation of S338/S339 and S330 (35, 36). Phosphorylation at S324/S325 is critical for the agonist-induced internalization of CXCR4, which is at least partially β-arrestin– and clathrin-dependent (37, 38).

It remains to be shown if C5aR1 and CXCR4 are direct targets of Norbin, and if Norbin affects their trafficking and signaling. One cannot predict if Norbin is able to interact with individual GPCRs, as there is no discernible sequence homology between its confirmed GPCR interactors. Here, we investigated the direct interaction of Norbin with C5aR1 and CXCR4 *in vitro*, and we used neutrophils from *Ncdn*^*Δmye*^ and *Ncdn*^*fl/fl*^ mice to investigate the interaction of the endogenous proteins in cells and the role of Norbin in the constitutive internalization, agonist-induced internalization and recycling of the endogenous receptors, and in neutrophil signaling. Our study provides new insights into the mechanisms through which Norbin controls GPCR trafficking and signaling.

## Results

### Norbin binds C5aR1 and CXCR4 and limits their cell surface levels in mouse neutrophils

We previously used mice with myeloid Norbin deficiency (*Ncdn*^*Δmye*^) to show that Norbin limits neutrophil-dependent antibacterial immunity and all neutrophil effector responses required for killing bacteria, particularly upon stimulation of GPCRs (12). We also found elevated levels of the GPCRs C5aR1 and CXCR4 on the surface of *Ncdn*^*Δmye*^ neutrophils compared to *Ncdn*^*fl/fl*^ control neutrophils, whereas the surface levels of two other GPCRs, CXCR1 and CXCR2, and of a range of adhesion molecules were normal (12). Here, we aimed to define the mechanisms by which Norbin controls the cell surface levels of C5aR1 and CXCR4.

**Figure 1:**
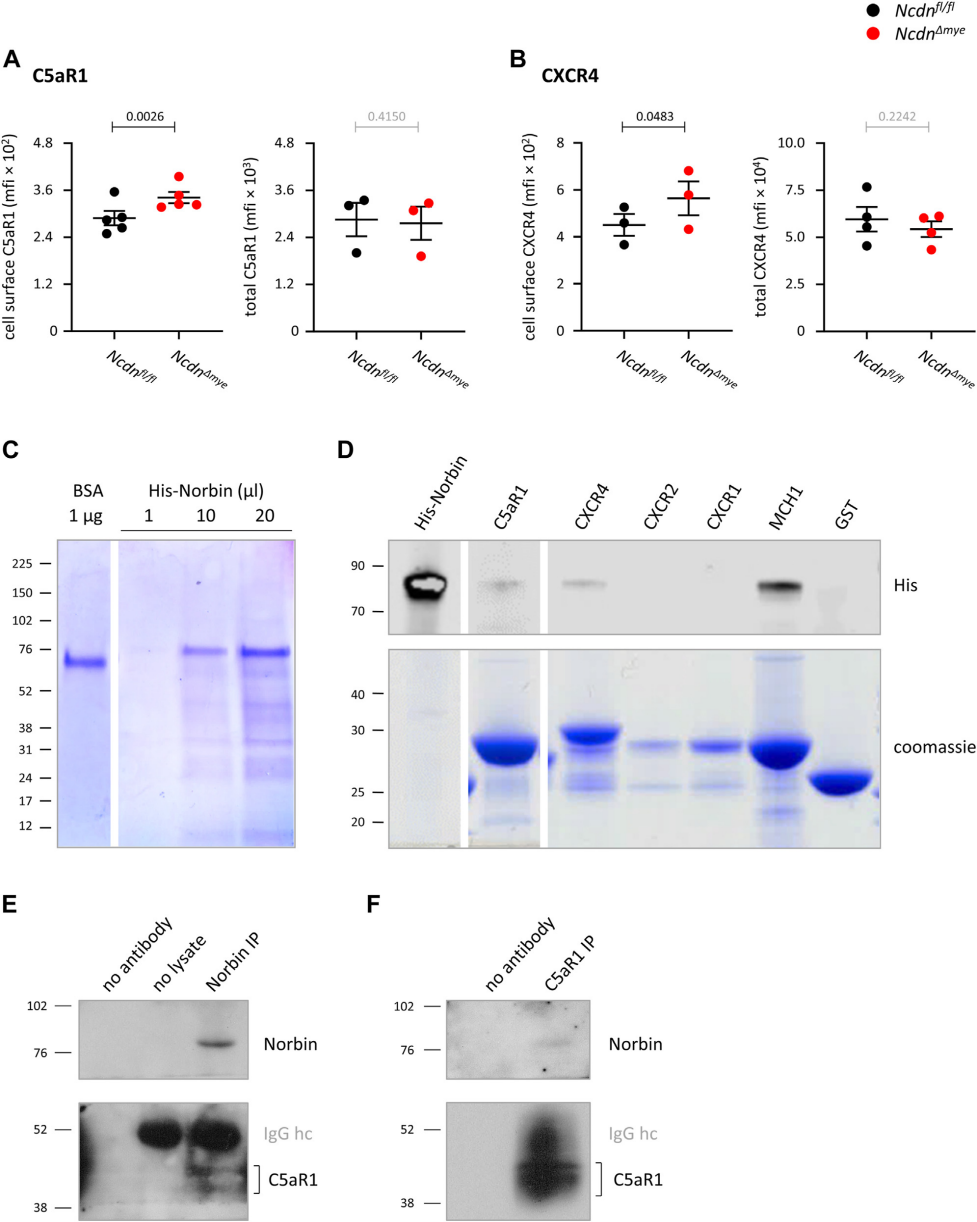
**Norbin binds C5aR1 and CXCR4 and limits their cell surface levels in mouse neutrophils.***A* and *B*, *Left-hand panels*: Cell surface levels of C5aR1 and CXCR4. Bone marrow cells from *Ncdn*^*fl/fl*^ (black) and *Ncdn*^*Δmye*^ (red) mice were stained on ice to identify live neutrophils (Mac1^hi^, Ly6G^hi^, FVD^lo^) and quantify the basal surface levels of (*A*) C5aR1 and (*B*) CXCR4 on the neutrophil surface by flow cytometry. Right-hand panels: Total levels of C5aR1 and CXCR4. Bone marrow cells were fixed, permeabilized, and stained to identify neutrophils markers (Mac1^hi^, Ly6G^hi^) and quantify the total levels of (*A*) C5aR1 and (*B*) CXCR4 in neutrophils by flow cytometry. Mean fluorescence intensities (mfi) were analyzed using FlowJo. Data are mean ± SEM of 3 to 4 independent experiments; each dot represents the mean of 1 experiment. Statistics are two-tailed paired Student’s *t* test. *p*-values in black show significant differences, *p*-values in *gray* are not significant. *C* and *D*, direct interaction of Norbin with C5aR1 and CXCR4 *in vitro*. Recombinant His-Norbin was purified (*C*) and incubated with the GST-tagged C-terminal tails of GPCRs, or GST, as indicated, and GST-tagged proteins were isolated using glutathione Sepharose (*D*). Samples were analyzed by SDS-PAGE and western blotting with His-tag antibodies (*upper panel*). Recombinant His-Norbin was loaded as a control. Coomassie staining (lower panel) shows the amount of GST-GPCR protein loaded. Representative blots shown are from 1 of 4 to 5 independent experiments. Direct binding of Norbin to C5aR1 and CXCR4 was seen in three out of five and four out of four experiments, respectively. *E* and *F*, endogenous Norbin and C5aR1 interact constitutively in neutrophils. Purified *Ncdn*^*fl/fl*^ neutrophils were lysed by sonication, and endogenous Norbin (*E*) or C5aR1 (*F*) were immunoprecipitated from the PGS. Controls without cell lysate or without immunoprecipitation antibody were processed in parallel as indicated. Protein interaction was analyzed by western blotting with antibodies against C5aR1 and Norbin. The heavy-chain of the immunoprecipitating antibodies (IgG hc) is indicated in *gray*. Blots shown are representative of 2 experiments.

First, we used flow cytometry to confirm the previously observed elevated cell surface levels of endogenous C5aR1 and CXCR4 in *Ncdn*^*Δmye*^ neutrophils (Fig. 1, *A* and *B*). In contrast, the total cellular levels of C5aR1 and CXCR4 were normal (Fig. 1, *A* and *B*), which suggested that Norbin may control the trafficking of C5aR1 and CXCR4.

Norbin can only control the trafficking of GPCRs that it interacts with directly, by binding to their intracellular C-terminal tails (8, 17, 18). To test if Norbin binds C5aR1 and CXCR4, we assessed the interaction between purified, recombinant His-Norbin and the glutathione-*S*-transferase (GST)-tagged C-terminal tails of human C5aR1 and CXCR4 *in vitro*. We used the GST-tagged C-terminal tail of MCH1 as a positive control, as MCH1 is a strong Norbin interactor (17, 18). We also tested CXCR1 and CXCR2, as CXCR1 and CXCR2 surface levels were normal in *Ncdn*^*Δmye*^ mouse neutrophils (12). Direct binding of His-Norbin was seen with C5aR1, CXCR4, and MCH1, whereas no binding was detected with CXCR1, CXCR2, or GST (Fig. 1, *C* and *D*). Hence, C5aR1 and CXCR4 are direct targets of Norbin.

In addition, we tested if Norbin and C5aR1 interact in cells. Endogenous C5aR1 coimmunoprecipitated with endogenous Norbin, and Norbin also coimmunoprecipitated with C5aR1, which shows that the proteins interact constitutively in primary neutrophils (Fig. 1, *E* and *F*).

To determine if Norbin controls the trafficking of C5aR1 and CXCR4 in mouse neutrophils, we assessed various GPCR trafficking processes, namely constitutive and agonist-induced receptor internalization, as well as the recycling of internalized receptors back to the cell surface.

### Norbin does not control the constitutive internalization of C5aR1 and CXCR4

**Figure 2:**
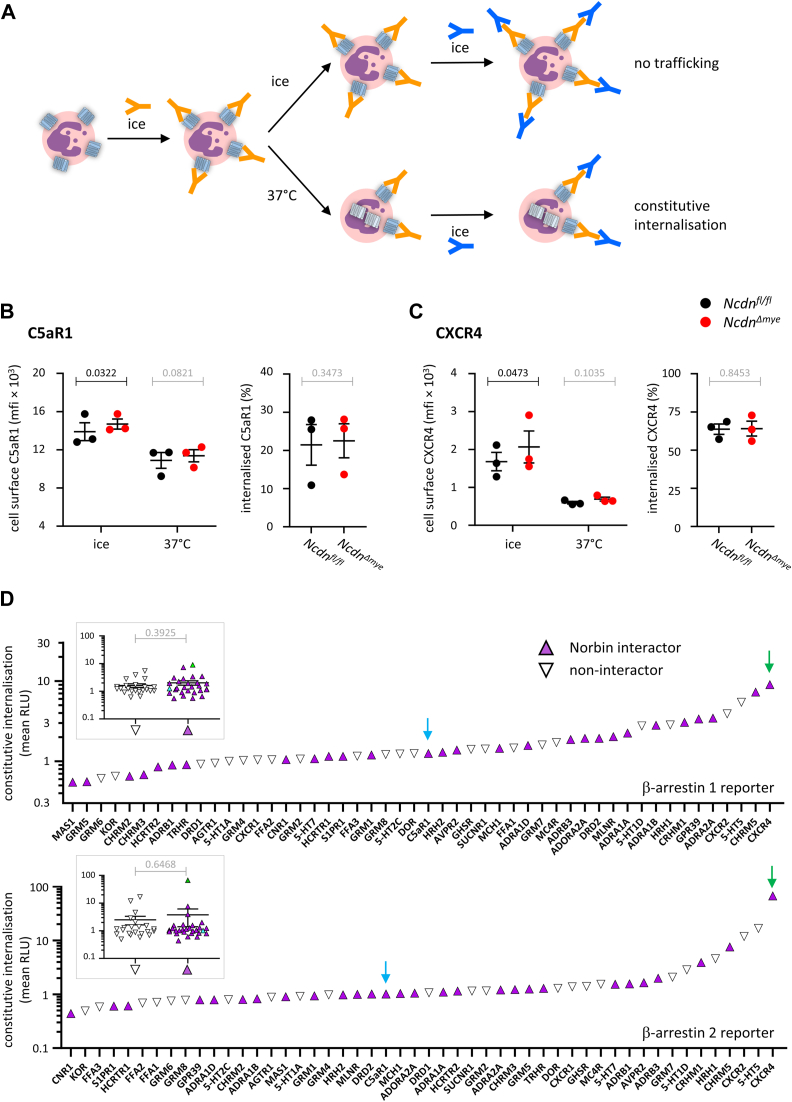
**Norbin does not control the constitutive internalization of C5aR1 and CXCR4.***A*, schematic of the constitutive receptor internalization assay. *B* and *C*, bone marrow cells from *Ncdn*^*fl/fl*^ (*black*) and *Ncdn*^*Δmye*^ (*red*) mice were stained on ice with primary antibodies to (*B*) C5aR1 or (*C*) CXCR4 to label the cell surface GPCRs. Cells were washed, and either kept on ice to prevent receptor trafficking, or incubated for 30 min at 37 °C to permit constitutive receptor internalization. Both sets of cells were then stained on ice with antibodies to identify live neutrophils (Mac1^hi^, Ly6G^hi^, FVD^lo^) and secondary antibodies to quantify the levels of C5aR1 or CXCR4 on the neutrophil surface by flow cytometry. *Left-hand panels*: mfi of C5aR1 or CXCR4 were analyzed using FlowJo. *Right-hand panels*: The constitutive internalization of C5aR1 or CXCR4 at 37 °C is expressed as % of the cell surface levels on ice. Data are mean ± SEM of three independent experiments; each dot is the mean of one experiment. Statistics in *left-hand panels* are two-way ANOVA with Šidák’s multiple comparisons test; *right-hand panels* are two-tailed paired Student’s *t* test. *p*-values in black show significant differences, *p*-values in *gray* are not significant. *D*, *in silico* analysis correlating the ability of Norbin to bind GPCRs to the relative constitutive rates of internalization of GPCRs known to be Norbin-interactors (*purple*) or non-interactors (*white*). GPCR trafficking data were extracted from the Tango-Trio database, which quantified β-arrestin 1 (*upper panel*) or β-arrestin 2 (*lower panel*) recruitment to constitutively active GPCRs. *Blue arrows* highlight the position of C5aR1 and green arrows CXCR4. The inserts show receptors grouped by interactors (*purple*) and non-interactors (*white*). Statistics are Student’s *t* test, *p*-values in *gray* are not significant.

GPCRs undergo constitutive internalization in the absence of ligand-binding, at varying rates (39, 40, 41, 42). To test if Norbin controls the constitutive internalization of endogenous C5aR1 and CXCR4 in neutrophils, we adapted a previously reported assay of constitutive GPCR internalization (43, 44) for use with flow cytometry. Bone marrow cells from *Ncdn*^*fl/fl*^ and *Ncdn*^*Δmye*^ mice were stained on ice with C5aR1 or CXCR4 antibodies, and then either kept on ice to prevent receptor trafficking or incubated at 37 °C to permit constitutive receptor internalization. Both sets of cells were then stained with secondary antibodies to quantify C5aR1 or CXCR4 on the neutrophil surface by flow cytometry (Fig. 2*A*). Both C5aR1 and CXCR4 underwent constitutive internalization, to varying degrees. Twenty two percent of C5aR1 and 68% of CXCR4 were internalized after 30 min. However, no differences in receptor internalization were observed between *Ncdn*^*Δmye*^ and *Ncdn*^*fl/fl*^ neutrophils (Fig. 2, *B* and *C*). Therefore, Norbin does not control the constitutive internalization of endogenous C5aR1 and CXCR4 in mouse neutrophils.

Effects of Norbin on the constitutive internalization of GPCRs have not been investigated before. To predict if Norbin controls the constitutive internalization of other GPCRs than C5aR1 and CXCR4, we performed an *in silico* analysis of the Tango-Trio database, which quantified the constitutive association of β-arrestin 1 and β-arrestin 2 with ∼350 human GPCRs as a measure of the constitutive activity of the receptors (45). As constitutive GPCR activity correlates closely with the constitutive rate of GPCR internalization (46), we could use the Tango-Trio dataset as a proxy for constitutive internalization and searched for correlations with the ability of Norbin to bind GPCRs. Including C5aR1 and CXCR4, 28 of the GPCRs tested in the database are known Norbin interactors, and 22 are known not to interact with Norbin (8, 17, 18). However, there was no correlation between the ability of Norbin to bind a GPCR and the inferred constitutive internalization rates of the receptors, regardless of whether the β-arrestin 1 or β-arrestin 2 based reporter systems were analyzed (Fig. 2*D*). Hence, Norbin does not affect the constitutive internalization of C5aR1 and CXCR4, and we predict that it will not control the constitutive internalization of its other GPCRs targets either.

### Norbin is required for the optimal agonist-induced internalization of C5aR1

Norbin was initially thought not to control the agonist-induced internalization of GPCRs because the agonist-stimulated internalization of MCH1 was unaffected by Norbin (17), but was recently shown to be required for the agonist-induced internalization of mGluR_1_ and mGluR_5_ in mouse hippocampal neurons (16), and of S1PR1 in neuronal PC12 cells (19). We tested here the role of Norbin in the agonist-induced internalization of endogenous C5aR1 and CXCR4 in mouse neutrophils.

**Figure 3:**
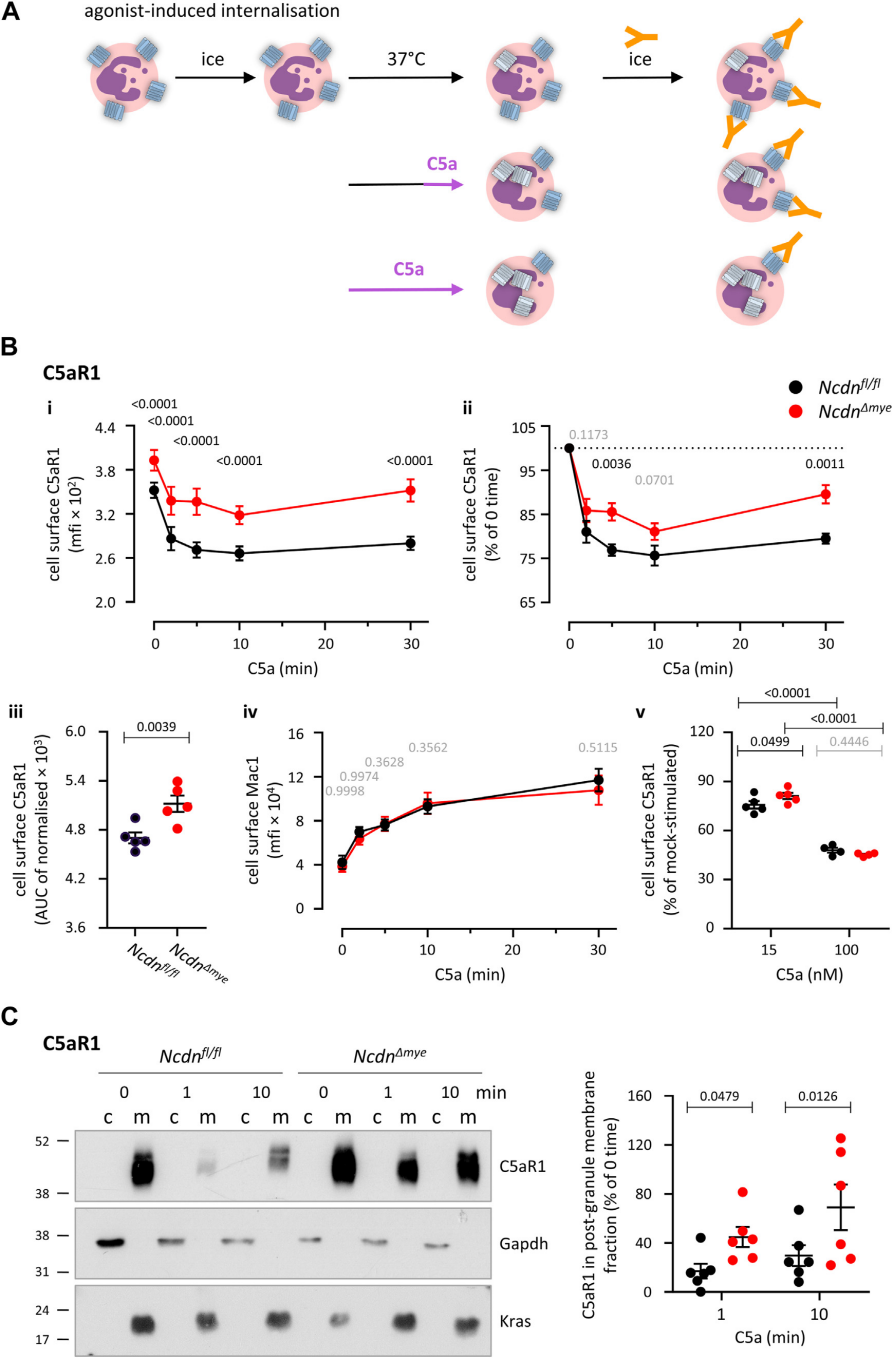
**Norbin is required fortheoptimal agonist-induced internalization of C5aR1.***A*, schematic of the agonist-induced receptor internalization assay. *B*, bone marrow cells from *Ncdn*^*fl/fl*^ (*black*) and *Ncdn*^*Δmye*^ (*red*) mice were incubated for 30 min at 37 °C. During this incubation, they were stimulated with 15 nM C5a for the indicated periods of time to induce the agonist-induced internalization of C5aR1. Cells were stained on ice to identify live neutrophils (Mac1^hi^, Ly6G^hi^, FVD^lo^) and quantify the level of C5aR1 on the neutrophil surface by flow cytometry. (i) The mfi of C5aR1 on the neutrophil surface was analyzed using FlowJo. (ii) To account for the increased basal cell surface level of C5aR1, data were normalized to 0 time. (iii) AUC of normalized cell surface C5aR1 over 30 min (iv) The mfi of Mac1 on the neutrophil surface was quantified as a control. (v) Level of C5aR1 on the neutrophil surface after incubation for 30 min at 37 °C, with either 15 nM or 100 nM C5a for the final 10 min of the incubation, as indicated. Data are mean ± SEM of five independent experiments (4 for 100 nM C5a, lower right); each dot is the mean of one experiment. Statistics are two-way ANOVA with Šidák’s multiple comparisons test, except (iii) which was two-tailed paired Student’s *t* test. *p*-values in black show significant differences, *p*-values in *gray* are not significant. *C*, cell fractionation. Purified *Ncdn*^*fl/fl*^ (*black*) and *Ncdn*^*Δmye*^ (*red*) neutrophils were incubated for 30 min at 37 °C. Some were stimulated with 50 nM C5a for 1 or 10 min towards the end of the 30 min incubation to induce the agonist-induced internalization of C5aR1. Cells were lysed in detergent-free buffer and fractionated by differential centrifugation. The post-granule supernatant (PGS) was ultracentrifuged to generate cytosol (c) and post-granule membrane (m) fractions which were analyzed by SDS-PAGE and western blotting with antibodies against C5aR1, Gapdh as cytosol marker, and Kras as plasma membrane marker. 20% of the plasma membrane and 1.4% of the cytosol fractions were loaded. The abundance of C5aR1 in the plasma membrane fraction was quantified by Fiji densitometry and adjusted for the Coomassie signal over the whole lane to correct for protein loading (see also Fig. 7). Representative blots are shown. C5aR1 in the membrane fraction is expressed as % of the 0-time control. Data are mean ± SEM of six independent experiments. Statistics are two-way ANOVA with Šidák’s multiple comparisons test.

To investigate the agonist-induced internalization of C5aR1, we stimulated bone marrow cells from *Ncdn*^*Δmye*^ and *Ncdn*^*fl/fl*^ mice with 15 nM C5a for various periods of time up to 30 min and assessed the level of C5aR1 on the neutrophil surface by flow cytometry (Fig. 3*A*). C5aR1 was internalized in response to C5a in both genotypes. However, significantly more C5aR1 remained at the cell surface in *Ncdn*^*Δmye*^ than in *Ncdn*^*fl/f*^ neutrophils throughout the time course (Fig. 3*B*i). To account for the constitutively enhanced surface C5aR1 level in *Ncdn*^*Δmye*^ neutrophils prior to C5a stimulation, we normalized the data to the 0-time controls, which revealed impaired C5a-stimulated internalization of C5aR1 in *Ncdn*^*Δmye*^ neutrophils throughout (Fig. 3*B*ii). Integration of the C5aR1 internalization response over the whole 30-min time course similarly demonstrated reduced agonist-induced internalization in *Ncdn*^*Δmye*^ neutrophils (Fig. 3*B*iii). Therefore, Norbin is required for the optimal agonist-induced internalization of C5aR1.

In basal neutrophils, some C5aR1 is stored on the membrane of secondary and tertiary granules, ready for upregulation to the plasma membrane during degranulation (26). We previously showed that *Ncdn*^*Δmye*^ neutrophils undergo some constitutive degranulation of tertiary granules (12). To assess if the impaired agonist-induced internalization of C5aR1 in *Ncdn*^*Δmye*^ neutrophils could be a consequence of enhanced degranulation bringing more C5aR1 to the plasma membrane, thereby reducing net loss of receptor from the surface, we assessed the β2 integrin Mac1, which is stored on the same granules (26). The surface level of Mac1 rose in response to C5a stimulation, as expected (12, 47). However, this was equal between *Ncdn*^*Δmye*^ and *Ncdn*^*fl/fl*^ throughout (Fig. 3*B*iv). Hence, the constitutive degranulation of tertiary granules does not underlie the reduced agonist-induced internalization of C5aR1 in *Ncdn*^*Δmye*^ neutrophils. Finally, we assessed the dose dependence of the impaired agonist-induced internalization, by comparing 15 nM C5a to 100 nM, which induces maximal internalization of C5aR1. At this high dose, the defect in agonist-induced internalization was overcome (Fig. 3*B*v), suggesting that Norbin is not absolutely required, and the internalization machinery is fundamentally intact.

To investigate the C5a-induced internalization of C5aR1 by another method than flow cytometry, we fractionated C5a-stimulated *Ncdn*^*Δmye*^ and *Ncdn*^*fl/fl*^ neutrophils by differential centrifugation (48). A 10,000*g* postgranule supernatant (PGS) was fractionated by ultracentrifugation into cytosol and postgranule membrane fractions, which were characterized by Gapdh as the cytosol marker and Kras as the plasma membrane marker. C5aR1 was exclusively found in the membrane fraction, as expected. In agreement with the flow cytometry results, C5aR1 was lost from the postgranule membrane fraction of *Ncdn*^*fl/fl*^ neutrophils upon C5aR1 stimulation, whereas significantly more C5aR1 was retained in this fraction in Ncdn^Δmye^ neutrophils (Fig. 3*C*). To verify that C5aR1 is trafficked from the plasma membrane into early endosomes upon C5a stimulation, we performed more detailed cell fractionation, by using Eea1 immunoprecipitation to isolate early endosomes from the PGS of C5a-stimulated *Ncdn*^*fl/fl*^ neutrophils prior to ultracentrifugation of the endosome-depleted PGS to obtain the plasma membrane fraction. C5aR1 was localized in the plasma membrane fraction prior to stimulation. The amount of C5aR1 decreased in the plasma membrane fraction and increased in the early endosome fraction upon C5a stimulation, peaking after 2 min. Ten minutes after C5a stimulation, C5aR1 was lower in both the plasma membrane and early endosome fractions, suggesting that the receptor was trafficked further into more mature endosomal compartments by that time (Fig. S1).

Together, these data indicate that Norbin is required for the optimal agonist-induced internalization of endogenous C5aR1 in neutrophils, in a dose-dependent manner, and this is not a consequence of altered neutrophil degranulation.

### Norbin does not control the agonist-induced internalization of CXCR4

**Figure 4:**
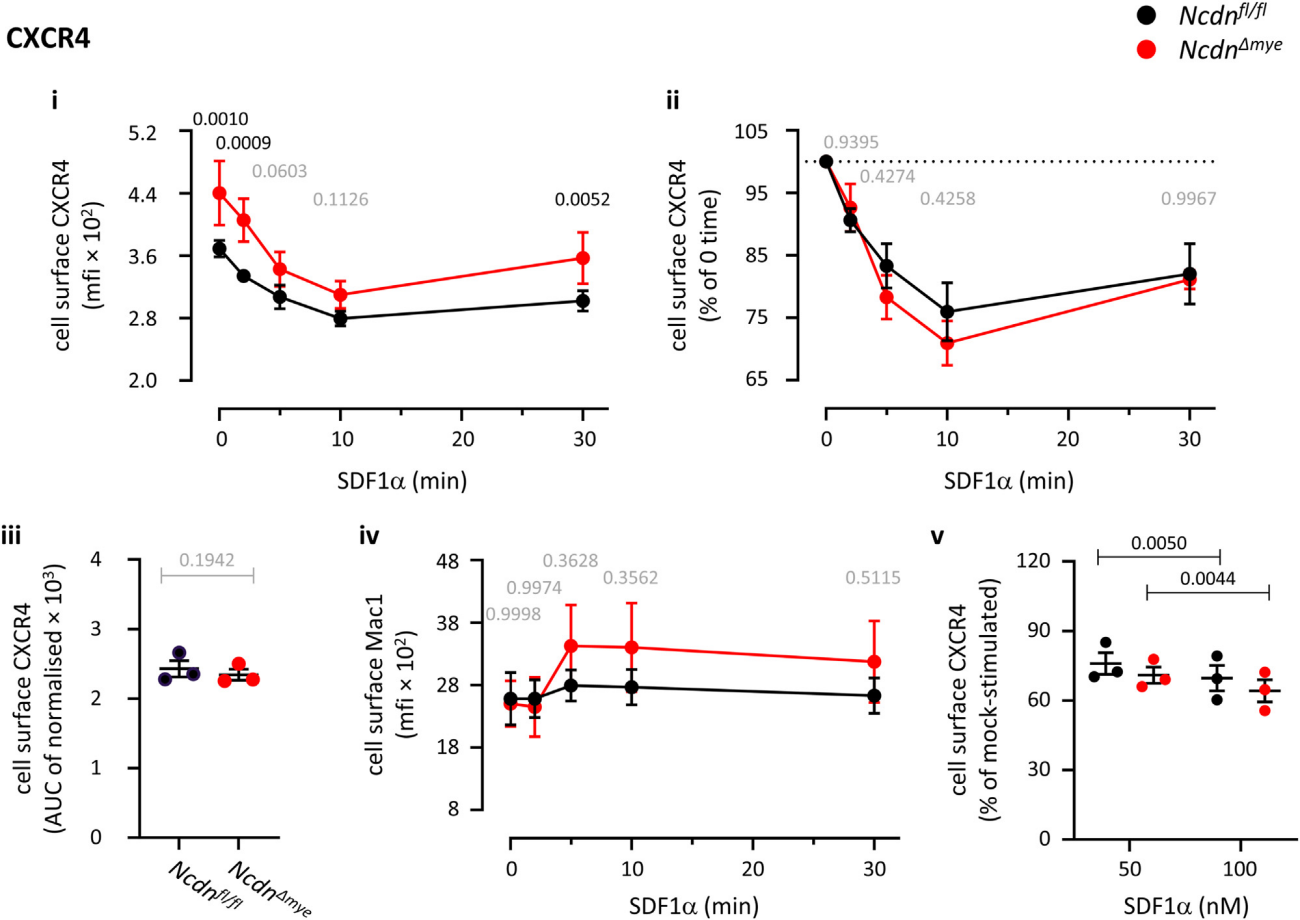
**Norbin does not controltheagonist-induced internalization of CXCR4.** Bone marrow cells from *Ncdn*^*fl/fl*^ (*black*) and *Ncdn*^*Δmye*^ (*red*) mice were incubated for 30 min at 37 °C. During this incubation, the cells were stimulated with 50 nM SDF1α for the indicated periods of time to induce the agonist-induced internalization of CXCR4. Cells were stained on ice to identify live neutrophils (Mac1^hi^, Ly6G^hi^, FVD^lo^) and quantify the level of CXCR4 on the neutrophil surface by flow cytometry. (i) The mfi of CXCR4 on the neutrophil surface was analyzed using FlowJo. (ii) To account for the increased basal cell surface level of CXCR4, data were normalized to 0 time. (iii) AUC of normalized cell surface CXCR4 over 30 min (iv) The mfi of Mac1 on the neutrophil surface was quantified as a control. (v) Level of CXCR4 on the neutrophil surface after incubation for 30 min at 37 °C including stimulation with either 50 nM or 100 nM SDF1α for the final 10 min of the incubation, as indicated. Data are mean ± SEM of 3 independent experiments; each dot is the mean of one experiment. Statistics are two-way ANOVA with Šidák’s multiple comparisons test, except (iv) which was two-tailed paired Student’s *t* test. *p*-values in black show significant differences, *p*-values in *gray* are not significant.

To investigate if Norbin controls the agonist-induced internalization of endogenous CXCR4 in neutrophils, we performed similar flow cytometry experiments as here-above, except stimulating the *Ncdn*^*Δmye*^ and *Ncdn*^*fl/fl*^ cells with 50 nM SDF1α. CXCR4 was internalized in response to SDF1α stimulation in both genotypes, and the surface level of CXCR4 remained higher in *Ncdn*^*Δmye*^ neutrophils throughout (Fig. 4i). However, normalizing to the 0-time control to account for the constitutively enhanced surface CXCR4 level in *Ncdn*^*Δmye*^ neutrophils revealed that CXCR4 was internalized in *Ncdn*^*Δmye*^ and *Ncdn*^*fl/fl*^ cells at the same rate in response to SDF1α stimulation (Fig. 4ii). Integration of the CXCR4 internalization response over the whole 30-min time course similarly demonstrated normal agonist-induced internalization of the receptor in *Ncdn*^*Δmye*^ neutrophils (Fig. 4iii). SDF1α stimulation induced less upregulation of Mac1 than C5a stimulation did, but again this was equal between the genotypes (Fig. 4iv). Furthermore, the lack of difference in CXCR4 internalization was not due to a saturating concentration of being SDF1α used, because 100 nM SDF1α induced a stronger internalization of CXCR4 in both genotypes. Therefore, Norbin does not control the agonist-induced internalization of CXCR4 in neutrophils, in contrast to C5aR1. This shows that Norbin regulates the trafficking of its target GPCRs through different mechanisms even within the same cell type.

### Norbin limits the recycling of internalized C5aR1 and CXCR4

After 30 min of agonist stimulation, surface levels of C5aR1 and CXCR4 began to increase again, and apparently more so in *Ncdn*^*Δmye*^ than *Ncdn*^*fl/fl*^ neutrophils (Figs. 3*B*ii and 4ii), which suggested that Norbin may regulate the recycling of internalized receptors back to the plasma membrane.

**Figure 5:**
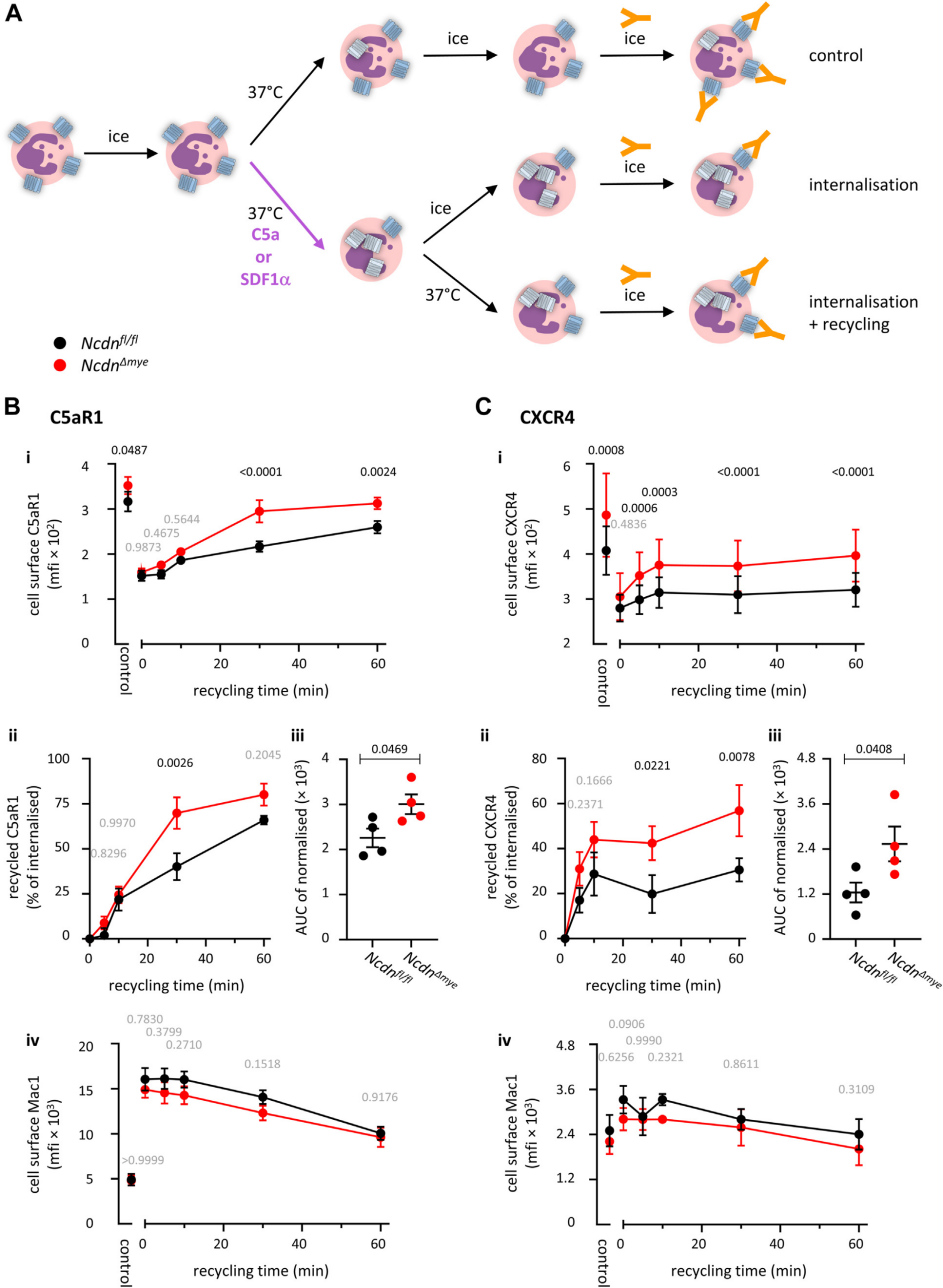
**Norbin limits the recycling of internalized C5aR1 and CXCR4 back to the cell surface.***A*, schematic of the receptor recycling assay. *B* and *C*, bone marrow cells from *Ncdn*^*fl/fl*^ (*black*) and *Ncdn*^*Δmye*^ (*red*) mice were stimulated for 10 min at 37 °C with high doses of agonist (*B*: 100 nM C5a; *C*: 100 ng/ml nM SDF1α) to induce the maximal internalization of C5aR1 and CXCR4, respectively, or were mock-stimulated. Cells were washed, and then either kept on ice as a control, or incubated at 37 °C for the indicated periods of time to allow the recycling of internalized receptors back to the cell surface and were then kept on ice. Cells were stained on ice to identify live neutrophils (Mac1^hi^, Ly6G^hi^, FVD^lo^) and quantify the level of (*B*) C5aR1 or (*C*) CXCR4 on the neutrophil surface by flow cytometry. (i) The mfi of C5aR1 and CXCR4 on the neutrophil surface was analyzed using FlowJo. (ii) To account for the increased basal cell surface level of the GPCRs, data were normalized by setting the maximally internalized level to 0 and basal receptor levels to 100%. (iii) AUC of normalized cell surface C5aR1 and CXCR4 over 60 min (iv) The mfi of Mac1 on the neutrophil surface was quantified as a control. Data are mean ± SEM of 4 independent experiments for each receptor; each dot is the mean of one experiment. Statistics are two-way ANOVA with Šidák’s multiple comparisons test, except middle right, two-tailed paired Student’s *t* test. *p*-values in black show significant differences, *p*-values in *gray* are not significant.

To investigate receptor recycling, we adapted an assay (49) using saturating concentrations of agonist to stimulate maximal internalization of cell surface GPCRs, followed by washing to remove the agonist, incubation at 37 °C to permit receptor recycling from endosomes back to the cell surface, and analysis of GPCR levels on the neutrophil surface by flow cytometry (Fig. 5*A*). To assess the recycling of C5aR1, *Ncdn*^*Δmye*^ and *Ncdn*^*fl/fl*^ cells were stimulated with 100 nM C5a for 10 min, and receptor recycling back to the cell surface was tracked over 60 min. The high concentration of C5a caused C5aR1 internalization to the same level in both genotypes, as expected, and C5aR1 surface levels rose again steadily throughout the recycling phase. However, cell surface C5aR1 recovered faster in *Ncdn*^*Δmye*^ than *Ncdn*^*fl/fl*^ neutrophils (Fig. 5*B*i). Normalization to the maximally internalized level before recycling confirmed increased recycling of C5aR1 back to the surface in *Ncdn*^*Δmye*^ neutrophils, with 70% recycling in *Ncdn*^*Δmye*^ neutrophils compared to 40% in *Ncdn*^*fl/fl*^ cells after 30 min (Fig. 5*B*ii). Integration of the recycling response over the 60-min time course also confirmed increased recycling in *Ncdn*^*Δmye*^ neutrophils (Fig. 5*B*iii). To verify that C5aR1 was delivered to the plasma membrane through the recycling route rather than through degranulation, Mac1 surface levels were again measured in parallel. Mac1 was upregulated to the cell surface upon C5a stimulation, as expected, and then declined steadily afterward. Importantly, Mac1 surface levels were similar between *Ncdn*^*Δmye*^ and *Ncdn*^*fl/fl*^ neutrophils throughout (Fig. 5*B*iv), which confirmed that the increased cell surface C5aR1 in *Ncdn*^*Δmye*^ neutrophils was not a consequence of degranulation but due to the enhanced recycling of internalized GPCR.

We measured the recycling of CXCR4 using the same approach, stimulating the cells with 100 ng/ml SDF1α and tracking the recycling of internalized CXCR4 back to the neutrophil surface over 60 min. CXCR4 was internalized to the same extent in *Ncdn*^*Δmye*^ and *Ncdn*^*fl/fl*^ neutrophils upon stimulation with the high concentration of agonist. CXCR4 surface levels rose again during the recycling phase, particularly within the first 10 min, but more so in *Ncdn*^*Δmye*^ than *Ncdn*^*fl/fl*^ neutrophils (Fig. 5*C*i–iii). Forty-two percent of the internalized receptor were recycled to the surface of *Ncdn*^*Δmye*^ neutrophils after 30 min compared to 20% in Ncdn^fl/fl^ cells (Fig. 5*C*ii). As a control, Mac1 was upregulated to the cell surface upon SDF1α stimulation, although less than in response to C5a, and then declined steadily, but its surface level was again identical between *Ncdn*^*Δmye*^ and *Ncdn*^*fl/fl*^ neutrophils throughout (Fig. 5*C*iv). Hence, Norbin limits the recycling of internalized C5aR1 and CXCR4 back to the neutrophil surface. This is the first report of a role of Norbin in receptor recycling.

### Norbin is required for β-arrestin recruitment in C5a-stimulated neutrophils

**Figure 6:**
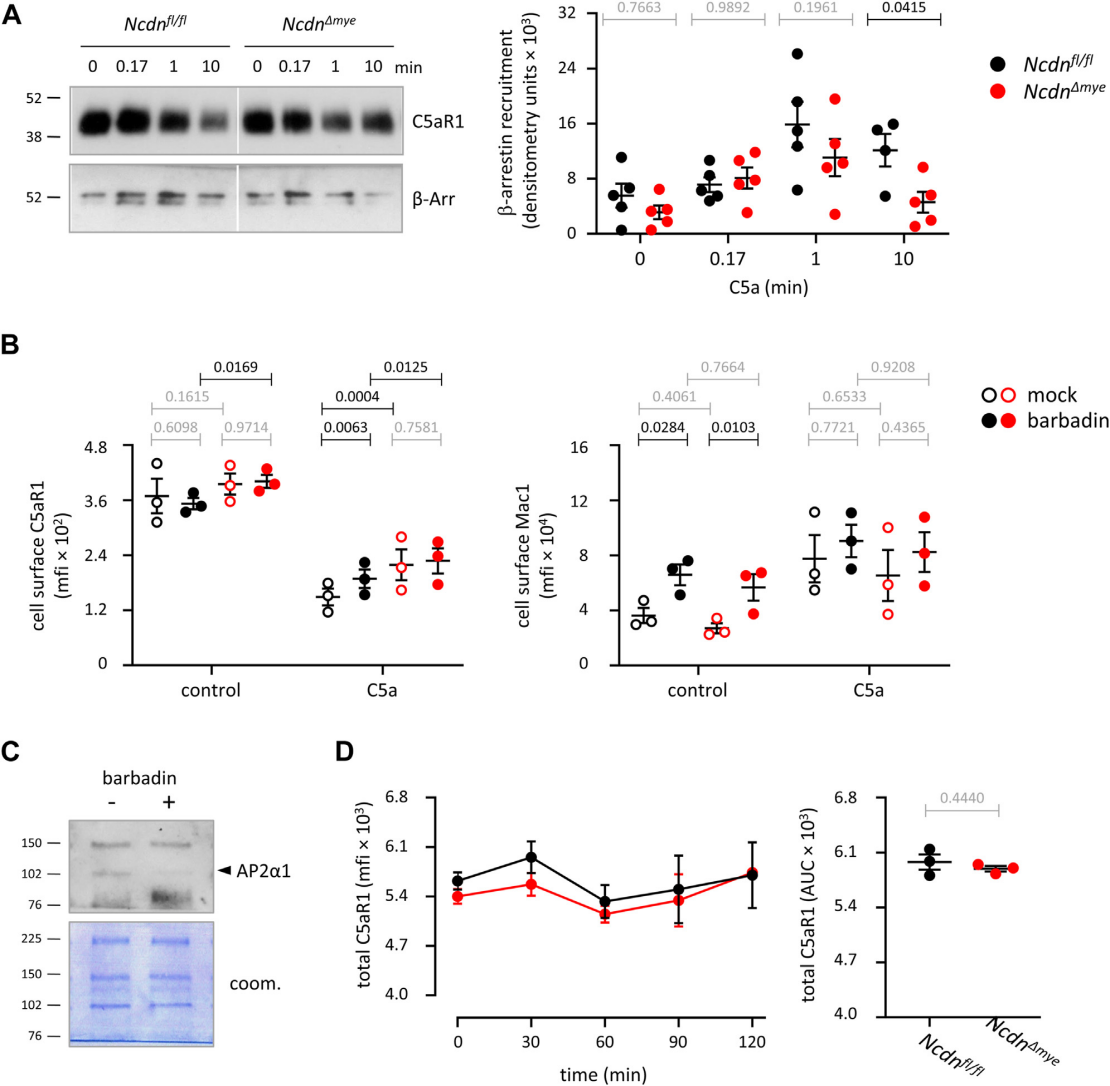
**Norbin is required for sustained β-arrestin recruitment and promotes the agonist-induced internalization of C5aR1 in a β-arrestin-dependent manner.***A*, β-arrestin recruitment. Purified *Ncdn*^*fl/fl*^ (*black*) and *Ncdn*^*Δmye*^ (*red*) neutrophils were incubated for 10 min at 37 °C while being stimulated with 15 nM C5a for 0, 0.17, 1 or 10 min, as indicated. Cells were lysed, a post-granule supernatant (PGS) was prepared by centrifugation, and glycosylated proteins, including C5aR1, were purified from the PGS using wheat-germ agglutinin agarose. Proteins were analyzed by SDS-PAGE and western blotting with C5aR1 and β-arrestin (β-Arr) antibodies. Representative western blots are shown. Blots were quantified by Fiji densitometry, and the β-arrestin signal was normalized to the amount of C5aR1 in each lane. Data are mean ± SEM of five independent experiments; each dot is the mean of one experiment. Statistics are two-way ANOVA with Šidák’s multiple comparisons test. *p*-values in black show significant differences, *p*-values in *gray* are not significant. *B*, β-arrestin-dependent agonist-induced internalization. Bone marrow cells were pretreated with 100 μM barbadin for 30 min at 37 °C (*filled symbols*), or mock-treated with 1% DMSO (*open symbols*), and stimulated for 5 min with 50 nM C5a, or mock-stimulated (control). Cells were stained on ice to identify live neutrophils (Mac1^hi^, Ly6G^hi^, FVD^lo^) and quantify the level of C5aR1 on the neutrophil surface by flow cytometry. The mfi of C5aR1 and Mac1 on the neutrophil surface were analyzed using FlowJo. Data are mean ± SEM of 3 independent experiments; each dot is the mean of one experiment. Statistics are two-way ANOVA with Šidák’s multiple comparisons test. *p*-values in black show significant differences, *p*-values in *gray* are not significant. *C*, barbadin blocks the recruitment of the clathrin-adaptor AP2. Purified *Ncdn*^*fl/fl*^ neutrophils were incubated for 30 min at 37 °C in the presence of 100 μM barbadin or mock-treated, prior to stimulation with 15 nM C5a for 1 min at 37 °C. Glycosylated proteins purified from the PGS using wheat-germ agglutinin agarose as in (*A*) and analyzed by western blotting with AP2α1 antibody. Coomassie staining was used as a loading control. The blot shown is representative of two independent experiments. *D*, receptor degradation. Purified neutrophils were incubated for 2 h at 37 °C while being stimulated with 15 nM C5a for the indicated periods of time towards the end. Cells were fixed, permeabilized, and stained to analyse the total cellular level of C5aR1 by flow cytometry. *Left-hand panel*: The mfi of total neutrophil C5aR1 was analyzed using FlowJo. *Right-hand panel*: AUC of the 2 h time course. Data are mean ± SEM of three independent experiments; each dot is the mean of one experiment. Statistics are two-tailed paired Student’s *t* test. *p*-values in *gray* are not significant.

Binding of β-arrestin to activated GPCRs promotes receptor internalization (50) and restricts receptor recycling back to the plasma membrane (51, 52). Given the importance of β-arrestin in both agonist-induced internalization and recycling, we assessed C5a-induced β-arrestin recruitment in *Ncdn*^*Δmye*^ and *Ncdn*^*fl/fl*^ neutrophils. Available antibodies were unsuitable for coimmunoprecipitating endogenous β-arrestin with endogenous C5aR in primary neutrophils. Hence, we made use of the fact that C5aR1, like most GPCRs, is heavily glycosylated (53). We enriched glycosylated proteins from the PGS of C5a-stimulated neutrophils by wheat-germ agglutinin affinity purification, before immunoblotting for C5aR1 and β-arrestin. Some β-arrestin was bound prior to C5a stimulation, potentially reflecting constitutive β-arrestin binding to GPCRs (54), but this basal β-arrestin binding was even between *Ncdn*^*Δmye*^ and *Ncdn*^*fl/fl*^ neutrophils (Fig. 6*A*). Upon stimulation with 15 nM C5a, β-arrestin was recruited within 10 s in *Ncdn*^*fl/fl*^ neutrophils, peaked at 1 min, and declined again somewhat by 10 min. In contrast, β-arrestin recruitment was more transient in *Ncdn*^*Δmye*^ neutrophils, returning to basal levels within 10 min (Fig. 6*A*). To investigate if Norbin affects both β-arrestin 1 and β-arrestin 2, we quantified the two isoforms separately. Norbin was required for the optimal recruitment of both β-arrestin isoforms, with no apparent preference (Fig. S2). Hence, Norbin is required for sustained β-arrestin recruitment in response to C5a stimulation. The reduced β-arrestin recruitment may explain the impaired agonist-induced internalization and accelerated recycling of C5aR1 in *Ncdn*^*Δmye*^ neutrophils.

### Norbin promotes the agonist-induced internalization of C5aR1 in a β-arrestin–dependent manner

To investigate further the role of β-arrestin in the C5a-stimulated internalization of C5aR1, we used barbadin, a small molecule inhibitor of the interaction between β-arrestin and its effector, the β2 subunit of the clathrin adaptor protein AP2 (55). Bone marrow cells from *Ncdn*^*Δmye*^ and *Ncdn*^*fl/fl*^ mice were treated with barbadin, or mock-treated, prior to C5a stimulation and analysis of C5aR1 cell surface levels by flow cytometry. In mock-treated cells, C5aR1 was internalized upon C5a stimulation, and this internalization was impaired in *Ncdn*^*Δmye*^ neutrophils, as expected (Fig. 6*B*). Barbadin treatment reduced the C5a-dependent internalization of C5aR1 in *Ncdn*^*fl/fl*^ neutrophils but not in Ncdn^Δmye^ cells (Fig. 6*B*). These data suggest that Norbin promotes the agonist-induced internalization of C5aR1 in a β-arrestin–dependent manner.

Barbadin is known to have a priming effect on neutrophils (56). We confirmed this by showing that barbadin treatment causes the upregulation of Mac1 to the surface of mock-stimulated cells. However, the priming occurred to the same extent in *Ncdn*^*Δmye*^ and *Ncdn*^*fl/fl*^ neutrophils and was overcome by C5a stimulation (Fig. 6*B*). Therefore, the priming effect of barbadin did not influence the agonist-induced internalization of C5aR1. To verify that barbadin has on-target effects in neutrophils, we treated neutrophils with barbadin, or mock-treated them, prior to C5a stimulation and isolation of glycosylated proteins from the PGS using wheat-germ agglutinin as here-above. Western blotting confirmed that barbadin inhibited the recruitment of the β-arrestin effector AP2α1 (Fig. 6*C*). Therefore, we can conclude that Norbin promotes the agonist-induced internalization of C5aR1 in neutrophils in a β-arrestin–dependent manner.

Prolonged exposure of cells to GPCR agonists can lead to GPCR degradation (57), which led us to monitor the total cellular level of C5aR1 in neutrophils stimulated with 15 nM C5a for up to 2 h using flow cytometry. C5aR1 levels were stable under these conditions and were similar in *Ncdn*^*Δmye*^ and *Ncdn*^*fl/fl*^ neutrophils throughout (Fig. 6*D*). Therefore, Norbin does not obviously affect the degradation of C5aR1.

To summarize the role of Norbin in the regulation of C5aR1 and CXCR4 trafficking in mouse neutrophils: Norbin is required for the agonist-induced internalization of C5aR1 through a β-arrestin–dependent mechanism, and it limits the recycling of internalized C5aR1 and CXCR4 back to the cell surface. Norbin does not control the agonist-induced internalization of CXCR4, nor does it affect the constitutive internalization of C5aR1 or CXCR4. These trafficking roles of Norbin result in increased levels of C5aR1 and CXCR4 on the neutrophil surface.

### Norbin limits the C5a-stimulated recruitment of Tiam1, Vav1, and PKCδ to the plasma membrane, and constitutively suppresses Vav activity

**Figure 7:**
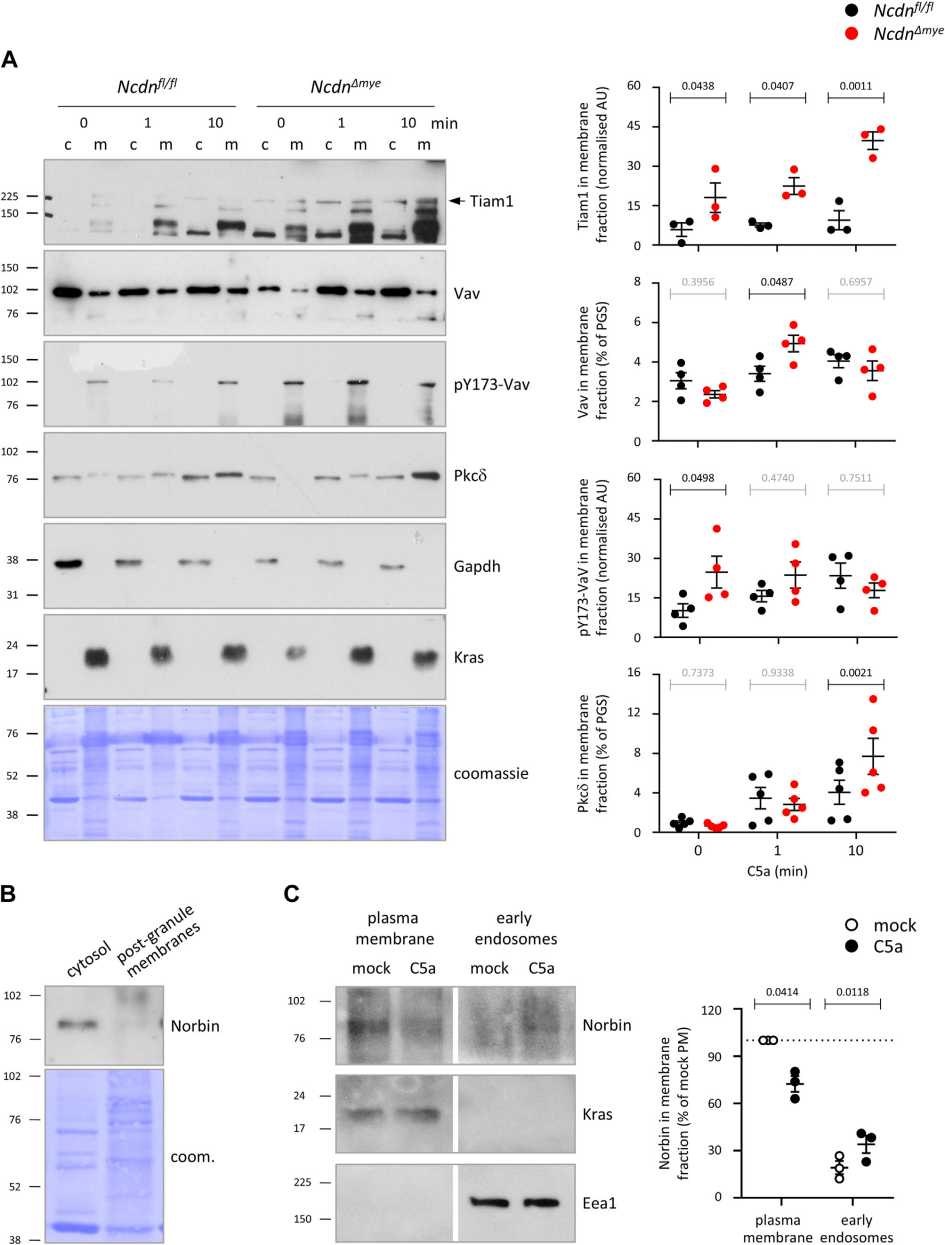
**Norbin limits the C5a-stimulated recruitment of Tiam1, Vav1 and PKCδ to the plasma membrane, andsuppressesthe constitutive activity of Vav.***A*, (i) Cytosol (c) and post-granule membrane (m) fractions of C5a- or mock-stimulated *Ncdn*^*fl/fl*^ (*black*) and *Ncdn*^*Δmye*^ (*red*) neutrophils from the same experiments as shown in Fig. 3*C* were analyzed by SDS-PAGE and western blotting with antibodies against Tiam1, Vav, active phospho-Y173-Vav, and PKCδ. 20% of the membrane and 1.4% of the cytosol fractions were loaded. Blots showing the cytosol marker Gapdh and plasma membrane marker Kras are included for reference. Membranes were Coomassie-stained to control for protein loading. (ii) Blots were quantified by Fiji densitometry. Tiam1 and Vav in cytosol and membrane fraction were quantified as % of the PGS. phospho-Y173-Vav and PKCδ in the membrane fraction were normalized to the Coomassie signal over the whole lane. Data are mean ± SEM of 3 to 5 independent experiments, as indicated; each dot is the mean of one experiment. Statistics are two-way ANOVA with Šidák’s multiple comparisons test. *p*-values in black show significant differences, *p*-values in *gray* are not significant. *B*, norbin is largely cytosolic. Cytosol and post-granule membrane fractions from *Ncdn*^*fl/fl*^ neutrophils were western blotted for Norbin. 20% of the plasma membrane and 1.4% of the cytosol fractions were loaded. *C*, norbin translocates from the plasma membrane into early endosomes upon C5a stimulation. *Ncdn*^*fl/fl*^ neutrophils were stimulated for with 100 nM C5a for 2 min (n = 2) or 10 min (n = 1), or were mock-stimulated, and early endosomes were isolated from the PGS using Eea1 immunoprecipitation before the plasma membrane was purified from the endosome-depleted PGS by ultracentrifugation. The fractions were western blotted for Norbin, Kras and Eea1 as indicated. The same cell-equivalents of plasma membrane and endosome fractions were loaded. Representative blots shown are from 1 of 3 independent experiments. Norbin blots were quantified by Fiji densitometry. Date are mean and ± SEM of 3 independent experiments. Statistics are two-way ANOVA with Šidák’s multiple comparisons test.

We previously showed elevated activation of Erk and the small GTPase Rac in *Ncdn*^*Δmye*^ compared to *Ncdn*^*fl/fl*^ neutrophils stimulated with fMLP, and constitutive activity of the Rac-GEF Vav (12). To investigate if the effects of Norbin on C5aR1 trafficking may translate into increased C5a signaling pathway activity, we first used the cytosol and membrane fractions of purified *Ncdn*^*fl/fl*^ and *Ncdn*^*Δmye*^ neutrophils from the experiments shown in Fig. 3*C* to assess the membrane localization of the Rac-GEFs Tiam1 and Vav, and of the protein kinase PKCδ, as plasma membrane translocation of these proteins is correlated with their activity (58, 59, 60). The membrane localization of Tiam1 was constitutively increased in *Ncdn*^*Δmye*^ compared to *Ncdn*^*fl/fl*^ neutrophils, and stimulation with C5a for 1 or 10 min caused a further progressive increase of Tiam1 in the membrane fraction of *Ncdn*^*Δmye*^ neutrophils that was not seen in *Ncdn*^*fl/fl*^ cells (Fig. 7*A*). The level of Vav membrane association was equal between the genotypes in mock-stimulated cells, and rose upon C5a stimulation in both genotypes, but more so in *Ncdn*^*Δmye*^ neutrophils, with a peak at 1 min (Fig. 7*A*). PKCδ localization in the membrane fraction was low in mock-stimulated cells and rose upon C5a stimulation in both genotypes, but again more so in *Ncdn*^*Δmye*^ neutrophils at 10 min (Fig. 7*A*). We also evaluated the level of the active form of Vav, which is phosphorylated on tyrosine 173 (58), in the plasma membrane fraction. Active, phospho-Y173-Vav was exclusively found in the membrane fraction, and as we previously showed, Vav was constitutively active in *Ncdn*^*Δmye*^ compared to *Ncdn*^*fl/fl*^ neutrophils (Fig. 7*A*). Therefore, Norbin suppresses the C5a-stimulated membrane translocation of Tiam1, Vav, and PKCδ, which is indicative of the activation of these signaling proteins, as well as constitutively limiting Vav activity. Together, with the GPCR trafficking data, these findings elucidate mechanisms through which Norbin suppresses GPCR-mediated responses in mouse neutrophils.

Norbin is a largely cytosolic protein, with only a small proportion being membrane-localized in a range of cell types (1, 5). To evaluate the subcellular localization of Norbin in neutrophils, we western blotted cytosol and postgranule membrane fractions, which confirmed that Norbin is largely cytosolic (Fig. 7*B*). To investigate furthermore if the membrane-localized portion of Norbin is at the plasma membrane and is trafficked into early endosomes upon C5a stimulation, we prepared early endosome and plasma membrane fractions similar to those shown in Fig. S1 for C5aR1. Norbin was present in the plasma membrane fraction, and some translocated into the early endosome fraction upon stimulation with C5a (Fig. 7*C*). Therefore, the membrane-associated portion of Norbin localizes to similar compartments as C5aR1, suggesting partial colocalization and internalization into endosomes.

### Norbin suppresses Erk and p38 Mapk signaling and Gα_i_-dependent ROS production in C5a-stimulated neutrophils

**Figure 8:**
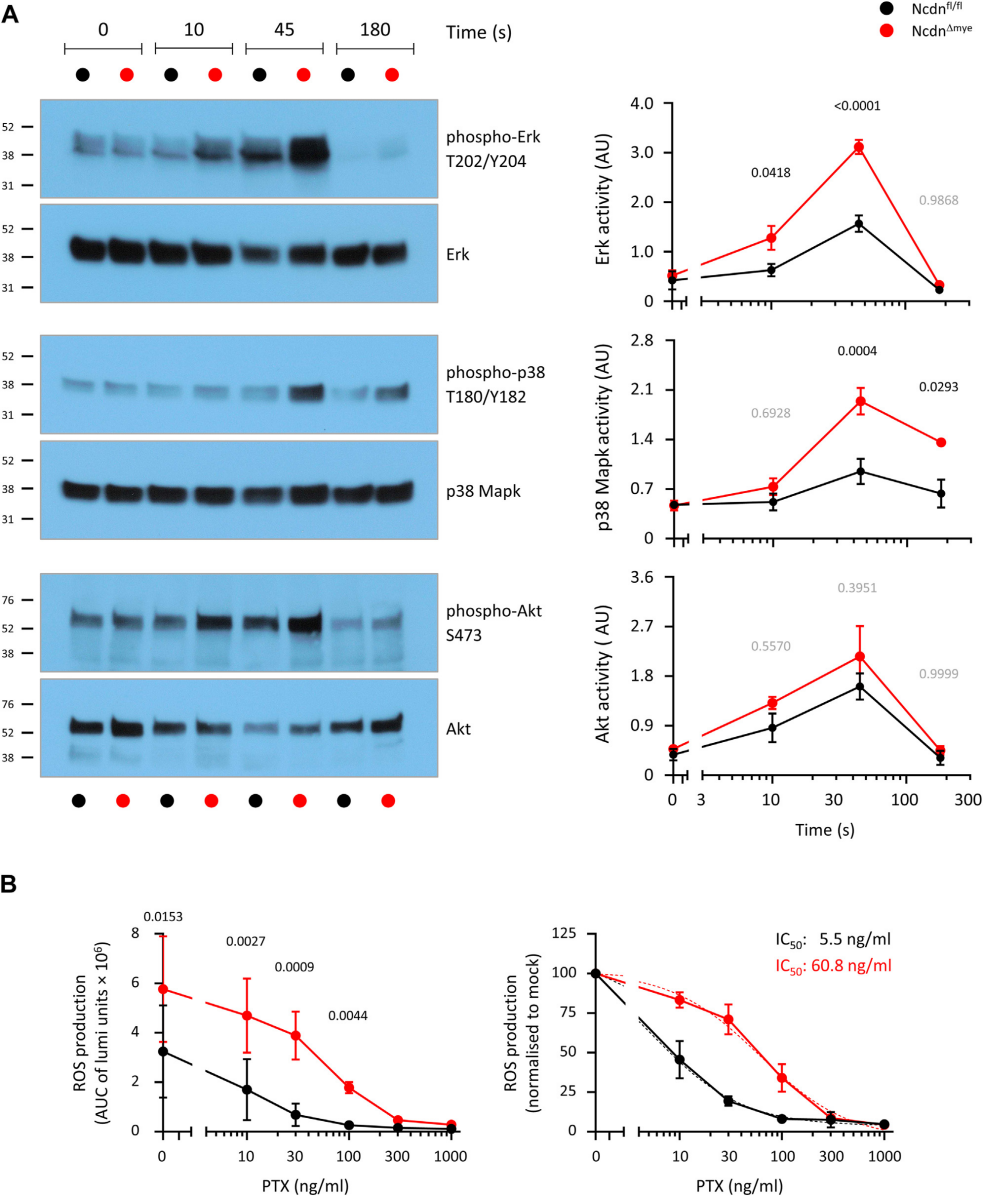
**Norbin suppresses Erk and p38 Mapk signaling and Gα**_**i**_**-dependent ROS production in C5a-stimulated neutrophils.***A*, Norbin suppresses Erk and p38 Mapk signaling. *Ncdn*^*fl/fl*^ (*black*) and *Ncdn*^*Δmye*^ (*red*) neutrophils were stimulated with 15 nM C5a as indicated, lysed, and analyzed by western blotting for phosphorylated, active and total Erk, p38 Mapk and Akt. Representative blots are shown. Quantification by ImageJ densitometry of phospho-signals divided by total protein. Data are mean ± SEM of three independent experiments. Statistics are two-way ANOVA with Šidák’s multiple comparisons test. *p*-values in black show significant differences, *p*-values in *gray* are not significant. *B*, norbin suppresses Gα_i_-dependent ROS production. ROS production in the presence of pertussis toxin (PTX) was measured in purified *Ncdn*^*fl/fl*^ (*black*) and *Ncdn*^*Δmye*^ (*red*) neutrophils using real-time chemiluminescence assay. Neutrophils were pre-incubated with increasing concentrations of PTX, as indicated, primed with TNFα/GM-CSF, and stimulated with 25 nM C5a. (*left*) ROS production was quantified by integrating the area under the curve (AUC) of the ROS response over 5 min and plotted as a function of the PTX concentration. Date are mean and ± SEM of three independent experiments. Statistics are two-way ANOVA with Šidák’s multiple comparisons test. *p*-values showing significant differences are indicated. (*right*) Data were normalized to the mock-treated condition for each genotype, and best-fit curves (*dotted lines*) and IC_50_s were determined using GraphPad.

To investigate the importance of Norbin for C5a-stimulated neutrophil signaling more generally, we tested Erk, p38 Mapk and Akt signaling in *Ncdn*^*fl/fl*^ and *Ncdn*^*Δmye*^ neutrophils stimulated with 15 nM C5a by western blotting total lysates for the phosphorylated active and total levels of these proteins. All three pathways were activated under these conditions, with a peak at 45 s. However, in *Ncdn*^*Δmye*^ neutrophils, Erk and p38 Mapk activities were elevated compared to *Ncdn*^*fl/fl*^ cells, whereas Akt showed a trend to increased activity but was normal overall (Fig. 8*A*). Therefore, Norbin suppresses Erk and p38 Mapk signaling, but not Akt signaling in C5a-stimulated neutrophils.

Finally, we previously showed that Norbin suppresses C5a-stimulated ROS production in neutrophils (12). To test whether this is due to lower activity of the Gα_i_ proteins that couple to the C5aR1, we measured C5a-stimulated ROS production in *Ncdn*^*Δmye*^ and *Ncdn^fl/fl^* neutrophils in the presence of pertussis toxin (PTX), which inhibits the interaction of Gα_i_-coupled GPCRs with Gα_i_ by ADP-ribosylating the G protein. We used an assay which measures the production of both extracellular and intracellular ROS in real time. *Ncdn*^*Δmye*^ neutrophils showed around 2-fold more ROS production in response to C5a stimulation, as expected (12), but 12 times more PTX was required to inhibit the ROS response by 50% in *Ncdn*^*Δmye*^ neutrophils than in *Ncdn^fl/fl^* neutrophils (Fig. 8*B*). Thus, Norbin limits Gα_i_-dependent ROS production in C5a-stimulated neutrophils.

Together, our signaling analysis showed that Norbin limits the membrane translocation and activation of a wide range of signaling mediators in C5a-stimulated neutrophils.

## Discussion

We showed that Norbin interacts directly with C5aR1 and CXCR4, identifying these GPCRs as Norbin targets, and we demonstrated that Norbin controls the trafficking of endogenous C5aR1 and CXCR4 in mouse neutrophils, limiting their cell surface levels. Norbin mediates the agonist-induced internalization of C5aR1 through a β-arrestin–dependent mechanism and limits the recycling of internalized C5aR1 and CXCR4 back to the cell surface. Norbin does not control the constitutive internalization of C5aR1 and CXCR4, nor does it affect the agonist-induced internalization of CXCR4. Norbin suppresses C5aR1 signaling in mouse neutrophils by limiting the C5a-stimulated membrane translocation of Tiam1, Vav, and PKCδ which is required for activity, as well as limiting the C5a-stimulated activation of the Erk and p38 Mapk pathways and Gα_i_-dependent ROS production. Overall, Norbin acts as a suppressor of C5aR1 and CXCR4 function.

We observed direct binding of recombinant His-Norbin with C5aR1 and CXCR4 *in vitro* and constitutive interaction of endogenous Norbin and C5aR1 in neutrophils, whereas CXCR1 and CXCR2 did not bind Norbin. Accordingly, Norbin controls the cell surface levels of C5aR1 and CXCR4 but not CXCR1 and CXCR2 in mouse neutrophils (this paper and (12)). This is consistent with other studies showing that Norbin interacts constitutively in cells with GPCRs that it binds directly *in vitro*, and conversely that it does not coimmunoprecipitate with or affect the trafficking or signaling of GPCRs that it cannot bind directly (8, 14, 16, 17, 18). His-Norbin bound C5aR1 and CXCR4 less than MCH1, so it would be interesting to assess binding affinities in the future, for example, by surface plasmon resonance. One could also generate C5aR1 and CXCR4 mutants with altered affinity for Norbin, to determine effects on GPCR trafficking, signaling and cell responses. Similarly, one could attempt to mutate Norbin to alter its affinity for target GPCRs. Notably, an A687G mutation was recently serendipitously found to block the interaction of Norbin with mGluR5 (16). However, it is difficult to devise Norbin mutants in a rational manner, as the protein consists almost entirely of α-helices and is predicted to adopt a boomerang shape ideal for protein–protein interactions over a large surface area (1), and mutations are prone to disrupting such structures.

We used cell fractionation to assess the subcellular localization of Norbin in neutrophils, by isolating early endosomes using Eea1 immunoprecipitation and preparing plasma membrane and cytosol from the endosome-depleted fraction. We show that Norbin is largely cytosolic in neutrophils, with only a small portion localized at membranes, whereas C5aR1 was of course entirely membrane-bound. The largely cytosolic localization of Norbin was expected from studies on Norbin localization in other cell types (5, 6, 61, 62). The membrane-bound portion of Norbin translocated from the plasma membrane to early endosomes upon C5a stimulation, as did C5aR1, consistent with the interaction of the two proteins in membranes. The trafficking of C5aR1 from the plasma membrane into early endosomes was very rapid, as expected from our flow cytometry assays, peaking in the early endosomal fraction after 2 min. Membrane-localized endogenous Norbin was near the detection limit, so we could only investigate either Norbin or C5aR1 in these experiments, not both. However, some Norbin translocation from the plasma membrane fraction into the endosomal fraction was seen upon C5a stimulation. Further investigation of C5aR1 and Norbin colocalization would need to be done in an overexpression system outside of primary neutrophils, which are terminally differentiated, short-lived (hours) cells that cannot be transfected.

The elevated steady-state levels of C5aR1 and CXCR4 on the surface of *Ncdn*^*Δmye*^ neutrophils mirror our recent finding that surface S1PR1 is constitutively raised in Norbin-deficient PC12 cells (19). In other contexts, Norbin expression, rather than Norbin deficiency, was found to increase the surface levels of MCH1, mGluR1, and mGluR5 (8, 16, 17). In all instances, the total cellular levels of the receptors were normal. Hence, Norbin regulates the steady-state surface levels of its target GPCRs through receptor trafficking rather than affecting the production or degradation of GPCRs, but the mechanisms through which Norbin controls steady-state GPCR levels vary and need to be determined on a receptor-by-receptor basis. Our analysis showed that Norbin does not control the constitutive internalization of C5aR1 and CXCR4. Similarly, our *in silico* analysis of the Tango-Trio dataset predicted that Norbin does not control the constitutive internalization of GPCRs in general. We tested both the β-arrestin 1 and β-arrestin 2 reporter datasets, as GPCRs have different binding preferences for these β-arrestin isoforms (63), but neither were correlated with Norbin binding. We measured constitutive receptor internalization by labeling cell-surface GPCR with anti-GPCR antibody prior to monitoring internalization at 37 °C. We should note that care must be taken not to use activating GPCR antibodies in such assays, as such antibodies will promote receptor endocytosis. We were careful to avoid the use of activating antibodies in this study. Considering that Norbin does not control the constitutive internalization of GPCRs generally, the altered steady-state levels are therefore more likely to be a consequence of different recycling rates of Norbin target GPCRs (see below).

In addition to regulating steady-state receptor trafficking, Norbin also promotes the agonist-induced internalization of some of its target GPCRs, including mGluR_1,_ mGluR_5_, and S1PR1 (16, 19), but not all, as the MCH-induced internalization of MCH1 was unaffected by Norbin expression in HEK293 cells (17). We showed here that Norbin promotes the C5a-stimulated internalization of C5aR1 but does not affect the SDF1α-induced internalization of CXCR4 in mouse neutrophils. Its role in the C5a-stimulated internalization of C5aR1 was concentration-dependent, seen at 15 nM but not 100 nM C5a, which demonstrated that Norbin promotes, but is not essential for, the agonist-induced internalization of C5aR1. However, 100 nM C5a may lie outside the physiologically relevant range, as the plasma C5a levels of endotoxaemic mice are an order of magnitude lower (20). The effect of Norbin on the agonist-induced internalization of C5aR1 was dependent on β-arrestin recruitment, as shown by impaired the C5a-stimulated internalization of the receptor upon barbadin treatment in *Ncdn*^*fl/fl*^ but not *Ncdn*^*Δmye*^ neutrophils. Therefore, we speculate that the ability of Norbin to promote the agonist-induced internalization of GPCRs depends on its role in β-arrestin recruitment, which may vary between GPCRs. As both Norbin and β-arrestin bind at the C-terminal tail of GPCRs, we speculate that the presence of Norbin sterically favors the binding of β-arrestin to the phosphorylated GPCR. The mechanism by which Norbin controls β-arrestin recruitment will require further investigation in the future, as our attempts to coimmunoprecipitate endogenous β-arrestin together with endogenous C5aR1 and Norbin were unsuccessful.

Norbin limited the recycling of internalized C5aR1 and CXCR4 back to the surface of mouse neutrophils, and as mentioned here-above, we speculate that recycling is the main mechanism through which Norbin controls the steady-state trafficking of its target GPCRs. It remains to be shown how Norbin controls GPCR recycling. One possibility is through its effects on β-arrestin. Binding of β-arrestin to activated GPCRs not only promotes receptor internalization (50), but it also inhibits GPCR recycling, by restricting the access of phosphatases to the phosphorylated receptor, thus preventing the dephosphorylation which is required for receptor recycling back to the plasma membrane (51, 52). Given our findings that Norbin governs the C5a-stimulated recruitment of β-arrestin and is required for the C5a-stimulated internalization of C5aR1, we postulate that its effect on β-arrestin recruitment may also govern its role in the recycling of internalized GPCRs. Another key regulator that promotes GPCR recycling is the retromer complex (64). It seems possible that Norbin might control recycling by preventing the interaction of retromer with C5aR1 and CXCR4. The retromer complex is a multiprotein complex, and its inhibitors are typically peptides and proteins which cannot be introduced into primary neutrophils. Therefore, this question will have to be addressed in the future in cell lines.

In basal neutrophils, the surface level of C5aR1 and CXCR4 is low, and both receptors are stored on the membrane of neutrophil granules (26). C5aR1, CXCR4, and other transmembrane receptors on granule membranes are upregulated by granule fusion with the plasma membrane during degranulation. The degranulation of secondary (lactoferrin) and tertiary (gelatinase) granules occurs during neutrophil priming, for example in response to stimulation with TNFα, granulocyte-macrophage colony-stimulating factor (GM-CSF), or lipopolysaccharide, as well as upon neutrophil activation (1, 12, 47, 65, 66). Hence, we needed to address the possibility that Norbin controls C5aR1 and CXCR4 trafficking by affecting the degranulation of secondary and tertiary granules. This was particularly relevant as we previously showed that basal *Ncdn*^*Δmye*^ neutrophils undergo some constitutive degranulation of tertiary granules, which was overcome by priming with TNFα and GM-CSF, or by stimulation with the chemotactic peptide fMLP (12). To control for degranulation, we assessed the β2 integrin Mac1, which, like C5aR1 and CXCR4, is stored on secondary and tertiary granules (26). Mac1 surface levels were even between *Ncdn*^*Δmye*^ and *Ncdn*^*fl/fl*^ under all conditions tested, suggesting that the roles of Norbin in neutrophil GPCR trafficking were not influenced by degranulation.

We previously hypothesized that the increased surface levels of GPCRs in *Ncdn*^*Δmye*^ neutrophils may underlie, at least in part, the elevated effector responses of *Ncdn*^*Δmye*^ neutrophils and improved neutrophil-dependent innate immunity of *Ncdn*^*Δmye*^ mice, by increasing the activity of the GPCR signaling pathways (12). We showed here that the increased surface levels of C5aR1 correlate with enhanced C5a-stimulated Tiam1, Vav, and PKCδ membrane localization in *Ncdn*^*Δmye*^ neutrophils, which equates to increased activity of these signaling pathways. In addition, we showed that Norbin limits the C5a-stimulated activation of Erk and p38 Mapk in neutrophils but does not affect Akt activity under the same conditions. Erk and p38 Mapk activation upon GPCR stimulation is linked to multiple neutrophil responses, notably with ROS production and migration, respectively (67), suggesting that Norbin suppresses these cell responses at least in part through the Erk and p38 MapK pathways. The fact that Akt activity was normal in Ncdn^Δmye^ neutrophils under the same conditions emphasizes that the increased cell surface level of a GPCR does not automatically result in increased signaling of all its downstream pathways, because intricate intracellular networks of control mechanisms influence signaling outcomes. To formally tease apart which signaling roles of Norbin are dependent on its underlying receptor trafficking roles one would need to generate Norbin mutants which permit one without the other, and as discussed here-above, Norbin structure does not lend itself to mutagenesis. The increased Tiam1 and Vav activities might explain the increased Rac activity we previously observed in in response to stimulation with fMLP (12), provided the fMLP receptor Fpr1 is also a Norbin target, which remains to be shown. We could not assess Fpr1 in the present study due to lack of suitable antibodies. Phosphorylation of C5aR1 at S334 is critical for the internalization of the activated receptor (29, 30). However, this phosphorylation is carried out by PKCβ (31) rather than PKCδ (31, 68). PKCδ is important in the neutrophil ROS response, as it phosphorylates the NADPH oxidase protein p40^phox^, thereby permitting the interaction of the signaling lipid phosphatidylinositol 3-phosphate with p40^phox^, and NADPH oxidase complex formation at the plasma membrane (69). Accordingly, we previously observed elevated C5a-induced ROS production in Ncdn^Δmye^ neutrophils (12). We showed here that substantially more PTX is required to inhibit C5a-stimulated ROS production in *Ncdn*^*Δmye*^ neutrophils than in *Ncdn*^*fl/fl*^ neutrophils, which suggested that Norbin limits Gα_i_-dependent cell responses upon the activation of C5aR1. Furthermore, while Norbin limits the constitutive cell surface levels and recycling of both C5aR1 and CXCR4, it promotes the agonist-induced internalization of C5aR1 but not CXCR4, which suggests that Norbin might suppress C5aR1 signaling more than CXCR4 signaling. Therefore, it would be interesting to study the effects of Norbin on neutrophil CXCR4 signaling in the future in a similar manner as done here for C5aR1.

Together, our study identifies C5aR1 and CXCR4 as Norbin targets and explains how Norbin suppresses the surface levels of these receptors and C5aR1 signaling in mouse neutrophils. It also furthers our understanding of the mechanisms through which Norbin regulates GPCR trafficking more generally, by identifying its importance in β-arrestin recruitment, β-arrestin dependent agonist-induced receptor internalization, and receptor recycling.

## Experimental procedures

### Mice

*Ncdn*^*Δmye*^ mice, which harbor a myeloid-lineage specific Norbin deficiency and were generated by crossing *Ncdn*^*fl/fl*^ (10) to *LysM*^*Cre*^ mice (70), were described previously (12). All mice were on C57BL/6 genetic background. To minimize genetic drift, the *Ncdn*^*fl/fl*^ and *Ncdn*^*Δmye*^ strains were intercrossed every 2 years. Mice were fed chow diet and water *ad libitum* and were group-housed (up to 5) in individually ventilated cages in the Babraham Institute Small Animal Unit which operates as detailed recently (47), using 12 h light/dark cycles with dusk and dawn settings. Cells isolated from young adult (8–14 weeks) *Ncdn*^*Δmye*^ and *Ncdn*^*fl/fl*^ mice of both sexes were used for experiments. Within experiments, mice were age- and sex-matched between genotypes. Animal breeding and experiments were carried out with approval from the local Animal Welfare Ethical Review Body under the British Home Office Animal Scientific Procedures Act 1986.

### Neutrophil isolation

Mature primary neutrophils were freshly purified from mouse bone marrow each day using a Percoll^PLUS^ gradient at 4 °C and endotoxin-free reagents, as previously described (65). Bone marrow cells were flushed from mouse femurs, tibias, and pelvic bones using ice-cold Hank’s balanced salt solution without Ca^2+^ or Mg^2+^ (HBSS^−−^, Sigma H6648) supplemented with 15 mM Hepes, pH 7.4 at room temperature (RT) (Sigma, H3784) and 0.25% fatty acid–free bovine serum albumin (BSA, Sigma, A8806) (HBSS^−−++^). The flushed bone marrow cells were triturated and filtered through 40 μm cell strainers. 58% isotonic Percoll^PLUS^ (GE Healthcare, 17544501) in HBSS^−−++^ was added as an underlayer, and samples were centrifuged at 1620*g* without brake for 30 min at 4 °C. The lower 3 ml were resuspended in 40 ml HBSS^−−++^ and centrifuged at 326*g* for 10 min at 4 °C. Erythrocytes were lysed using ice-cold Geye’s solution (130 mM NH_4_Cl, 5 mM KCl, 780 μM Na_2_HPO_4_, 176 μM KH_2_PO_4_, 5.5 mM glucose, 1 mM MgCl_2_, 280 μM MgSO_4_, 1.54 mM CaCl_2_, 13.4 mM NaHCO_3_) for 3 min on ice. Ten volumes of ice-cold HBSS^−−++^ were added, and the cells were centrifuged again. Neutrophils were resuspended in ice-cold Dulbecco’s PBS (DPBS) with Ca^2+^ and Mg^2+^ (Sigma, D8662) supplemented with 1 g/l glucose (Sigma, G8769) and 4 mM NaHCO_3_ (Sigma, S8761), all of tissue culture grade, (DPBS^++++^) and kept on ice. Aliquots were counted using an hemocytometer, and purity was assessed by Kwik-Diff staining (Thermo Scientific Shandon, 9990700) of cytospins. Preparations were typically 90 to 95% pure and yielded ∼1.6 × 10^7^ neutrophils per mouse. Neutrophils were sedimented, resuspended in the buffer appropriate for the subsequent assay, and kept on ice until use.

### Cell surface and total levels of GPCRs

Cell surface levels of GPCRs were measured by flow cytometry essentially as previously described (12, 19). Bone marrow cells were flushed from femurs, tibias, and pelvic bones using ice-cold HBSS^−−++^, filtered through 40 μm cell strainers, and counted by hemocytometer. Cells were pelleted at 326*g* for 10 min at 4 °C, resuspended in ice-cold DPBS^++++^ at 4 × 10^7^/ml, and kept on ice. Two hundred fifty microliters aliquots were spun at 10,000*g* for 30 s at 4 °C and blocked in 50 μl fluorescence-activated cell sorting (FACS) blocking buffer (DPBS^++++^, Fc block (BD Biosciences, Clone 2.4G2, 553142, 1:1000)) for 15 min on ice, prior to incubation for 20 min on ice in FACS blocking buffer containing the following cocktail to detect live neutrophils and cell surface GPCRs: fixable viability dye eFluor 780 (eBioscience, 65-0865-14, 1:1000), Cd11b-AF647 (BD Biosciences, clone M1/70, 557686, 1:1000) and Ly6G-BV421 (BioLegend, Clone 1A8, 127627, 1:500) antibodies, and either C5aR1-PE (Abcam, clone 20/70, 53434, 1:60) or CXCR4-PE (Invitrogen, clone 2B11, 12-9991-82, 1:60) antibodies. Cells were sedimented at 10,000*g* for 30 s, washed in 1 ml HBSS^−−++^, spun again, resuspended in 500 μl HBSS^−−++^, 1 mM EDTA, and analyzed by flow cytometry using a Bio-Rad ZE5 flow cytometer. 20,000 live neutrophils per sample were recorded by gating for fixable viability dye^lo^, CD11b^hi^, Ly6G^hi^ cells, and the mean fluorescence intensity of phycoerythrin (PE)-labeled GPCRs on the neutrophil surface was determined using FlowJo (www.flowjo.com).

Quantification of the total cellular levels of GPCRs was done in a similar manner, except in permeabilized cells. Bone marrow cells prepared as described here-above were resuspended in ice-cold DPBS^++++^ at 4 × 10^7^/ml, and 750 μl were mixed 1:1 with 4% paraformaldehyde in PBS, 1 mM EGTA, 0.5 mM MgCl_2_, and incubated for 15 min at RT. Cells were sedimented for 30 s at 10,000*g*, washed in 1 ml DPBS^++++^, spun again, and permeabilized in 500 μl DPBS^++++^, 0.5% Tween-20 for 10 min at RT. Cells were washed in DPBS^++++^, resuspended in 500 μl DPBS^++++^, and 125 μl aliquots were spun down, and blocked in 50 μl FACS blocking buffer for 15 min on ice. Cells were sedimented and incubated for 20 min with an ice-cold cocktail containing FACS blocking buffer with antibodies for neutrophil markers (Cd11b and Ly6G, as above), and either CXCR4-PE (as above) or C5aR1-biotin (Abcam, clone 10/92, ab54378, 1:20) antibodies. For C5aR1, cells were washed in DPBS^++++^ and stained in 50 μl FACS blocking buffer containing PE-streptavidin (BioLegend, 405203, 1:500). Stained cells were centrifuged at 10,000*g* for 30 s, washed in 1 ml HBSS^−−++^, spun down again, resuspended in 500 μl HBSS^−−++^, 1 mM EDTA, and analyzed by flow cytometry using a Bio-Rad ZE5 flow cytometer. Twenty thousand neutrophils per sample were recorded by gating for CD11b^hi^, Ly6G^hi^ cells, and the mean fluorescence intensity of total cellular PE-labeled GPCRs was quantified using FlowJo.

### Recombinant His-Norbin protein

A full-length rat Norbin construct with a C-terminal 6 × His epitope tag in pcDNA6 was used to produce His-tagged rat Norbin protein, which is 98% identical to human Norbin, essentially as previously described (17). Briefly, the construct was transformed into competent M15 [pREP4] bacteria (Gentaur, 820-S0027) by 42 °C heat-shock for 90 s, followed by a 1 h incubation in Psi broth at 37 °C with shaking. Bacteria were grown on LB agar plates with 25 μg/ml kanamycin and 100 μg/ml ampicillin selection. A single colony was grown in LB, 25 μg/ml kanamycin, 100 μg/ml ampicillin ON at 225 rpm, 37 °C, diluted 1:50 into 600 ml prewarmed LB with antibiotics, and grown to *A*_600_ 0.6 to 0.8, before induction with 0.1 mM IPTG for 22 h at 21 °C. Bacteria were pelleted, stored at −80 °C, and aliquots assessed for Norbin expression by western blotting with His antibody (Cell Signaling Technologies, 2365, 1:1000). The His-Norbin protein was largely insoluble in inclusion bodies, as expected, so it was renatured and purified essentially as described (18), with some modifications as detailed below.

Bacterial pellets were thawed on ice, resuspended in 40 ml ice-cold lysis buffer (10 mM imidazole, pH 8.0, 50 mM NaH_2_PO_4_, 300 mM NaCl, 25 μg/ml leupeptin, 25 μg/ml pepstatin A, 25 μg/ml aprotinin, 25 μg/ml pepstatin, 100 μM PMSF, and 1 μg/ml lysozyme (Thermo Fisher Scientific, 90082)), and incubated with end-over-end rotation for 1 h at 4 °C. The lysate was sonicated using a Q700 sonicator (QSonica) with a 1/2" tip at 60 amp for 9 × 20 s, with 100 s breaks on ice between each burst for cooling. The lysate was centrifuged at 10,000*g* for 30 min at 4 °C and the supernatant discarded. The pellet was resuspended in 20 ml ice-cold STE buffer (10 mM Tris–HCl, pH 8.0, 150 mM NaCl, 1 mM EDTA) and centrifuged at 10,000*g* for 30 min at 4 °C. This centrifugation/wash step was repeated twice more. The washed inclusion bodies were solubilized in 50 ml lysis buffer containing 6 M guanidine hydrochloride by sonication at 50 amp for 3 × 10 s, with 110 s breaks on ice between each burst for cooling. Samples were centrifuged at 15,000*g* for 20 min at 4 °C. The supernatant was transferred to dialysis tubing (Thermo Fisher Scientific, 21-152-9, prepared by rinsing for 2 h under running tap water, before extensive washing in distilled water) and allowed to dialyze ON at 4 °C against 1 l of 50 mM Hepes, pH 7.5, 400 mM L-arginine, 4 M guanidine hydrochloride, 1 mM EDTA, 1 mM DTT, 100 μM PMSF. 500 ml of the solution were removed and replaced with 500 ml 50 mM Hepes, pH 7.5, 400 mM L-arginine, 1 mM EDTA, 1 mM DTT, 100 μM PMSF. The sample was allowed to dialyze again ON at 4 °C, and this dialysis process was repeated until the guanidine hydrochloride concentration reached 1 M. The dialysis tubing was transferred into 1 l of 50 mM Hepes, pH 7.5, 400 mM L-arginine, 1 mM EDTA, 1 mM DTT, 100 μM PMSF. The L-arginine was replaced with 200 mM NaCl in two successive ON dialysis steps, using first 1 l of 50 mM Hepes, pH 7.5, 100 mM L-arginine, 50 mM NaCl, 1 mM EDTA, 1 mM DTT, 100 μM PMSF, and then then 1 l of 50 mM Hepes, pH 7.5, 200 mM NaCl, 1 mM EDTA, 1 mM DTT, 100 μM PMSF. The recovered His-Norbin solution was centrifuged at 15,000*g* for 20 min at 4 °C to remove insoluble material, and 1 ml aliquots were snap-frozen in liquid N_2_ and stored at −80 °C for single use. The protein concentration was estimated by SDS-PAGE and Coomassie staining compared to BSA protein standards.

### C-terminal peptides of GPCRs

Constructs encoding the glutathione S-transferase (GST)-tagged C-terminal tails of the GPCRs MCH1 and CXCR1 in pGEX4T3 were described previously (18). Constructs for the GST-tagged C-terminal tails of C5aR1 and CXCR2 in pGEX4T3 and CXCR4 in pGEX6P1 were produced by PCR amplification and subcloning into pGEX using BamH1 and Xho1. Constructs were designed to include GST and the entire C-terminus from 5 amino acids upstream of the conserved NPXXY sequence, *i.e*. INCCINPIIYVVAGQGFQGRLRKSLPSLLRNVLTEESVVRESKSFTRSTVDTMAQKTQAV for GST-C5aR1, FHCCLNPILYAFLGAKFKTSAQHALTSVSRGSSLKILSKGKRGGHSSVSTESESSSFHSS for GST-CXCR4, ANSCLNPFVYIVLCETFRKRLVLSVKPAAQGQLRAVSNAQTADEERTESKGT for GST-MCH1, LHSCLNPIIYAFIGQNFRHGFLKILAMHGLVSKEFLARHRVTSYTSSSVNVSSNL for GST-CXCR1, and LHSCLNPLIYAFIGQKFRHGLLKILAIHGLISKDSLPKDSRPSFVGSSSGHTSTTL for GST-CXCR2. Production of the GPCR C-terminal tail peptides was done essentially as described (18). Constructs were transformed into BL21 DE3 bacteria (NEB, C2527) and grown on LB agar plates with 100 μg/ml ampicillin. A single colony was grown in LB, 100 μg/ml ampicillin ON at 225 rpm, 37 °C, diluted 1:50 into 200 ml LB, 100 μg/ml ampicillin, and grown to *A*_600_ 0.7 to 0.9, before induction with 1 mM IPTG for 4 h at 37 °C. Cells were pelleted and stored at −80 °C. Pellets were thawed, resuspended in 20 ml STE buffer with 0.1 mg/ml lysozyme, and incubated with end-over-end rotation for 1 h at 4 °C. DTT and 10% sarcosyl in STE buffer were added to final 5 mM and 1.5%, respectively, and samples incubated with end-over-end rotation for 30 min at 4 °C. Lysates were sonicated using a Q700 sonicator (QSonica) with a half" tip at 50 amp for 5 × 20 s, with 100 s breaks on ice between each burst for cooling and centrifuged at 10,000*g* for 15 min at 4 °C. Protease inhibitors were added to the supernatant to final concentrations of 25 μg/ml leupeptin, 25 μg/ml pepstatin A, 25 μg/ml aprotinin, 25 μg/ml pepstatin, 100 μM PMSF, and 10% Triton X-100 in STE buffer was added to 2% final. The lysates were snap frozen in liquid N_2_ in 1 ml aliquots and stored at −80 °C for single use.

### Interaction of His-Norbin with the C-terminal peptides of GPCRs

The direct interaction of His-Norbin with the C-terminal peptides of GPCRs was tested *in vitro* essentially as previously described (18). Lysates containing the GST-tagged C-terminal peptides of GPCRs were thawed on ice. 400 to 1000 μl/sample were used, depending on protein concentration, and the volume of all samples was adjusted to 1 ml with PBS, 2% Triton X-100, 5 mM DTT (PBS-TD). Samples were precleared by incubation with 100 μl prewashed glutathione Sepharose (GE Healthcare, 17–0756–01) with end-over-end rotation for 3 h at 4 °C. Beads were washed twice in 1 ml PBS-TD and incubated in PBS-TD, 5 mM DTT, containing 50 μg purified recombinant His-Norbin with end-over-end rotation for 3 h at 4 °C. Samples were spun at 500*g* for 2 min, 4 °C and washed 3 × in 1 ml PBS, 1% Triton X-100. 50 μl prewarmed SDS-PAGE sample buffer was added, and proteins were eluted at 65 °C for 15 min before analysis by SDS-PAGE and western blotting using His antibody.

### Coimmunoprecipitation of Norbin and C5aR1

Purified neutrophils at 4 × 10^7^ cells/ml in ice-cold sonication buffer (150 mM NaCl, 50 mM HEPES pH 7.2, 5 mM EDTA, 25 μg/ml leupeptin, 25 μg/ml pepstatin A, 25 μg/ml aprotinin, 25 μg/ml antipain, 100 μM PMSF) were sonicated gently on ice using a microtip of the Misonix ultrasonicator XL on setting 2 (∼6 W), with 4 × 2 s bursts and 28 s cooling periods, so as not to disrupt their granules. Under these conditions, ∼5 to 10% of cells remained intact according to trypan blue exclusion assay. NP40 was added to 0.1% final concentration, and samples were incubated for 2 min on ice with frequent vortexing. Lysates were centrifuged at 14,000*g* for 10 min at 4 °C. The supernatant was incubated with a pool of Norbin antibodies (5, 6, 12) (C1, 1:500, N4, 1:1000, N7, 1:1000) or with C5aR1 antibody (7 TM antibodies, 7TM0032MN-IC, 1:200) for 2 h on ice with end-over-end rotation. Controls without lysate or without antibody were processed in parallel. 30 μl prewashed protein A magnetic beads (Pierce, 88846) were added, and samples incubated for 40 min on ice with end-over-end rotation. Beads were washed three times with sonication buffer before boiling in 1.3 × SDS-sample buffer. Samples were processed by western blotting, using blocking with 5% EasyBlocker (GeneTex, GTX425858) and incubations with Norbin (1:3000) or C5aR1 (1:1000) antibodies in EasyBlocker and EasyBlot anti-rabbit IgG-HRP secondary antibody (GeneTex, GTX225856–01, 1:1000 in EasyBlocker) to minimize the background from the immunoprecipitating IgGs.

### Constitutive GPCR trafficking

The constitutive internalization of GPCRs was adapted from (43, 44) except using flow cytometry as the end-point. Bone marrow cells prepared as described above were resuspended at 4 × 10^7^/ml in ice-cold DPBS^++++^, and 125 μl aliquots were transferred into precooled 1.7 ml tubes. Cells were centrifuged for 10 min at 326*g*, resuspended in 50 μl FACS blocking buffer, and blocked on ice for 15 min. Cells were centrifuged again, resuspended in FACS blocking buffer containing C5aR1-Biotin or CXCR4-Biotin (Invitrogen, clone 2B11, 12–9991–80, 1:60) antibodies, and incubated on ice for 30 min 1 ml ice-cold DPBS^++++^ was added, and cells were sedimented again and resuspended in 125 μl DPBS^++++^. Cells were either incubated 37 °C for the indicated periods of time to allow constitutive trafficking, and then transferred back onto ice, or were kept on ice throughout. Cells were pelleted for 30 s at 10,000*g* and resuspended in 50 μl FACS blocking buffer containing the antibody cocktail to detect viable neutrophils described here-above, and PE-streptavidin to detect C5aR1 or CXCR4. Samples were analyzed by flow cytometry for the levels of PE-labelled GPCRs on the neutrophil surface as described here-above.

### In silico analysis of constitutive GPCR trafficking

GPCRs which were known from the literature to be Norbin interactors, or not to bind Norbin, were analyzed *in silico* using the Tango-Trio data set, which employed a luciferase reporter system to quantify constitutive β-arrestin-1 or β-arrestin-2 recruitment to ∼350 GPCRs in the absence of agonist to determine the constitutive activities of these GPCRs (45). We used these constitutive β-arrestin binding data as a proxy for the relative rates of constitutive internalization of these GPCRs and interrogated them for correlations with the ability of Norbin to bind GPCRs.

### Agonist-induced internalization of GPCRs

The agonist-induced internalization of C5aR1 and CXCR4 was measured by flow cytometry essentially as previously described (12). Bone marrow cells prepared as described here-above were resuspended at 4 × 10^7^/ml in ice-cold DPBS^++++^, and 125 μl aliquots were transferred into precooled 1.7 ml tubes. Cells were incubated for 30 min at 37 °C and were then stimulated with the indicated concentrations of C5a (R&D systems, 2037-C5-025) or SDF1α (Peprotech, 250–20A) for the indicated periods of time, or were mock-stimulated. In some experiments, cells were pre-incubated with 100 μM barbadin in 1% DMSO for 30 min at 37 °C, or mock-treated with 1% DMSO, prior to stimulation with C5a. Samples were transferred onto ice to prevent further trafficking. Cells were spun down for 30 s at 10,000*g*, resuspended in 50 μl FACS blocking buffer, and blocked on ice for 15 min. Cells were sedimented again, stained with the antibody cocktail to detect viable neutrophils and C5aR1 or CXCR4, and analyzed by flow cytometry for the levels of PE-labelled GPCRs on the neutrophil surface as described here-above.

### Neutrophil fractionation

Neutrophils were fractionated by differential centrifugation essentially as previously described (48). Purified bone marrow-derived neutrophils were resuspended at 3.5 × 10^7^ cells/ml in DPBS^++++^. 1 × 10^7^ cells were incubated for 30 min at 37 °C, while being stimulated with 15 nM C5a for the indicated periods of time, or mock-stimulated. 1 ml ice-cold DPBS^++++^ was added, and cells were centrifuged for 30 s at 10,000*g* and resuspended in 1100 μl ice-cold detergent-free relaxation buffer (10 mM Pipes, pH 6.0; 100 mM KCl, 3 mM NaCl, 3.5 mM MgCl_2_, 1 mM EDTA, 1 mM ATP, 6 mM Na_3_VO_4_, 1 x PhosStop (Roche, 4906845001), 25 μg/ml leupeptin, 25 μg/ml pepstatin A, 25 μg/ml aprotinin, 25 μg/ml, 100 μM PMSF). Cells were gently sonicated on ice using a Misonix ultrasonicator XL with a microtip on setting 2 (∼6 W), with 5 × 2 s bursts and 28 s cooling periods. Under these gentle conditions, 5 to 10% of the cells remained viable according to trypan blue exclusion assay. 6 mM EGTA was added to the lysates, and samples were centrifuged for 10 min at 1000*g* to pellet nuclei, debris, and live cells, and obtain the post-nuclear supernatant (PNS). 1000 μl of the PNS were transferred into fresh pre-cooled tubes and centrifuged for 15 min at 10,000*g* to pellet granules. 950 μl of the post-granule supernatant (PGS) was transferred into precooled ultracentrifuge tubes and spun at 200,000*g* for 30 min at 4 °C. The supernatant, considered the cytosol fraction, was mixed 2:1 with boiling 4 × sample buffer and boiled for 5 min. The pellet, considered the post-granule membrane fraction, was washed once in relaxation buffer, resuspended in 100 μl boiling 1.3 × sample buffer, and boiled for 5 min. Samples were snap-frozen in liquid N_2_ and stored at −80 °C before analysis by SDS-PAGE using 12% gels and western blotting with antibodies against the cytosol marker Gapdh (ProteinTech, 60004-1-Ig, 1:20,000) and the plasma membrane marker Kras (ProteinTech, 12063-1-AP, 1:6000) and against endogenous C5aR1 (7 TM, 7TM0032MN-IC, 1:1000), or the signaling proteins described below. Membranes were stained with Coomassie to control for protein loading. Protein abundance in each fraction was quantified by densitometry of blots using Fiji (ImageJ).

In some experiments, early endosomes were isolated from the PGS by Eea1 immunoprecipitation before the plasma membrane fraction was obtained from the endosome-depleted PGS using a method was adapted from (71). The PGS was prepared as described here above, except from three times more cells per sample, stimulated with or without 100 nM C5a, transferred into fresh, precooled tubes, and whole endosomes were immunoprecipitated by incubation with Eea1 antibody (Cell Signaling Technology 3288, 1:1000) for 1 h on ice with end-over-end rotation. Prewashed Pierce protein A magnetic beads (ThermoFisher Scientific, 88846) were added, and samples were incubated for a further 40 min. The beads containing immunoprecipitated endosomes were pelleted using magnets and washed 3 × in lysis buffer. The endosome-depleted PGS was transferred into precooled ultracentrifuge tubes and ultracentrifuged at 200,000*g* for 30 min at 4 °C. The pellet, considered the plasma membrane fraction, was washed once by careful addition and aspiration of relaxation buffer. Boiling 1.3 × SDS-PAGE sample buffer was added to both endosomal and plasma membrane fractions, and samples were boiled for 5 min and then snap-frozen in liquid N_2_. Samples were analyzed by western blotting with Norbin (C1, 1:1000), C5aR1, Eea1 (Cell Signaling Technology 3288, 1:3000), and Kras antibodies, using EasyBlocker and EasyBlot secondary antibodies as described here-above.

### GPCR recycling

The recycling of internalized GPCRs back to the plasma membrane was adapted from (49), except using flow cytometry as the end-point. Bone marrow cells prepared as described here-above were resuspended in ice-cold DPBS^++++^ at 4 × 10^7^/ml, and 125 μl aliquots were transferred into precooled 1.7 ml tubes. Cells were stimulated with high doses of C5a (100 nM) or SDF1α (100 ng/ml) for 10 min at 37 °C to induce maximal receptor internalization, or were mock-stimulated. 1 ml ice-cold DBPS^++++^ was added, and cells were sedimented at 300*g* for 10 min at 4 °C. Cells were resuspended in 125 μl ice-cold DPBS^++++^ and incubated again at 37 °C for the indicated periods of time to allow the recycling of internalized receptors back to the plasma membrane, before being transferred back onto ice. Cells were spun down for 30 s at 10,000*g*, resuspended in 50 μl FACS blocking buffer, and blocked on ice for 15 min. Cells were sedimented again, stained with the antibody cocktail to detect viable neutrophils and C5aR1 or CXCR4, and were analyzed by flow cytometry for levels of PE-labelled GPCRs on the neutrophil surface as described here-above.

### β-arrestin recruitment

To measure the C5a-stimulated recruitment of β-arrestin, we used affinity purification with wheat-germ agglutinin to enrich glycosylated proteins (72), which include GPCRs (73). Purified bone marrow-derived neutrophils were resuspended at 2.75 × 10^7^ cells/ml in DPBS^++++^. 5.5 × 10^6^ cells were incubated for 10 min at 37 °C while being stimulated with 15 nM C5a for the indicated time, or mock-stimulated. 0.8 ml ice-cold 1.2 × lysis buffer (60 mM Tris, pH 7.5, 180 mM NaCl, 1.2% Nonidet P-40, 0.12% SDS, 0.6% sodium deoxycholate, 6 mM EDTA, 6 mM Na_4_P_2_O_7_, 6 mM Na_3_VO_4_, 6 mM β-glycerophosphate, 25 μg/ml leupeptin, 25 μg/ml pepstatin A, 25 μg/ml aprotinin, 25 μg/ml, 100 μM PMSF) was added, and samples were incubated for 3 min on ice with frequent vortexing. In some experiments, cells were pre-incubated with 100 μM barbadin (Merck, SML3127, in 1% DMSO) for 30 min at 37 °C, or mock-treated with DMSO, prior to C5a stimulation. Lysates were centrifuged at 14,000*g* for 10 min at 4 °C, and the post-granule supernatant (PGS) incubated on a rotator for 1 h at 4 °C with 30 μl wheat-germ agglutinin agarose (Vector Laboratories, AL-1023, prewashed in lysis buffer). Beads were washed 3 × with 1 ml lysis buffer, and proteins were eluted by incubation in 1.3 × SDS-PAGE sample buffer at 50 °C for 20 min. Proteins were analyzed by SDS-PAGE and western blotting with C5aR1, β-arrestin 1/2 (Cell Signaling Technologies, 4674, 1:500) and AP2α1 (Proteintech, 29887-1-AP, 1:1000) antibodies. Blots were analyzed by Fiji densitometry, and β-arrestin levels were normalized to the C5aR1 signal for each condition.

### GPCR degradation

Purified bone marrow-derived neutrophils were resuspended at 4 × 10^7^ cells/ml in DPBS^++++^. 1 × 10^7^ cells were incubated for 2 h at 37 °C while being stimulated with 15 nM C5a for the indicated periods of time, or mock-stimulated. Cells were prevented from settling by gently flicking tubes every 30 min. Samples were mixed 1:1 with 4% paraformaldehyde (2% final concentration) and incubated for 15 min at RT. Cells were centrifuged at 10,000*g* for 30 s, washed in HBSS^−−++^, sedimented again, and incubated in permeabilization/blocking (PB) buffer (DPBS^++++^, 5% BSA, 0.1% saponin) for 15 min at RT. Cells were analyzed by flow cytometry for the total cellular level of PE-labelled C5aR1 in neutrophils as described above.

### Signaling pathway analysis

In some experiments, the membrane localisation of signaling proteins was analyzed. Purified neutrophils were stimulated with C5a and fractionated as described here-above, and the cytosol and post-granule membrane fraction were western blotted for Tiam1 (Bethyl Laboratories, A300–099A, 1:1000), Vav (Dr Martin Turner, Babraham Institute, 1:1000), phospho-Y173-Vav (ProSci (79–457, 1:1000), and PKCδ (Cell Signaling Technology, 2058, 1:1000). Western blots were quantified by ImageJ densitometry. The amounts of Tiam1 and Vav in the cytosol and membrane fraction were calculated as a percentage of the PGS. The amounts of phospho-Y173-Vav and PKCδ in the plasma membrane fraction were normalized to the Coomassie signal over the whole lane. In other experiments, signaling pathway analysis was done on whole cell lysates. Purified neutrophils were resuspended in ice-cold DPBS^++++^ at 1 × 10^7^/ml, and 150 μl aliquots were prewarmed for 5 min at 37 °C before stimulation with 50 μl of 60 nM C5a (4 × ) for various periods of time. The reaction was stopped by the addition of 1 ml ice-cold DBPS^++++^, and the cells were sedimented at 10000*g* for 30 s at 4 °C. Cells were lysed in 150 μl ice-cold RIPA buffer (30 mM Hepes, pH 7.4, 150 mM NaCl, 1% Nonidet P-40, 0.5% deoxycholate, 0.1% SDS, 5 mM EGTA, 4 mM EDTA) supplemented with 1 mM DTT, protease inhibitors (100 μM PMSF, 25 μg/ml each of leupeptin, pepstatin-A, aprotinin and antipain), and phosphatase inhibitors (50 mM NaF, 10 mM β-glycero-phosphate, 3 mM sodium orthovanadate, and 5 mM sodium pyrophosphate) for 5 min on ice with frequent vortexing. 75 μl boiling 4 × SDS-sample buffer were added and samples boiled for 10 min before snap-freezing in liquid nitrogen. Samples were western blotted using primary antibodies from Cell Signaling Technology (London, UK), comprising phospho-Akt S473 (9271, 1:500), Akt (9272, 1:1000), phospho-p38 Mapk (9211, 1:500), p38 Mapk (9212, 1:1000), phospho-Erk1/2 (4370, 1:4000), Erk1/2 (9102, 1:2000) and secondary goat anti-rabbit IgG (Bio-Rad, 1706515, 1:3000). Detection was done using Clarity ECL reagent (Bio-Rad, 1705060, 1:3000). Blots for phosphorylated proteins were done first, then stripped, and reprobed for the total proteins. The activities of Akt, p38 Mapk and p44/42 Erk were quantified using Fiji densitometry, and the phospho-signals divided by the total for each protein.

### ROS production

ROS production was measured by luminol chemiluminescence assay in a Berthold MicroLumat Plus luminometer (Berthold Technologies), essentially as described (12). Purified neutrophils were resuspended at 5 × 10^6^ cells/ml in DPBS^++++^ and 1.25 × 10^6^ cells were treated with various concentrations of PTX (PTX, Sigma, 516560, in DPBS^++++^) for 3.5 h at 37 °C, or mock-treated in DPBS^++++^. 45 min before the end of the PTX treatment, murine TNFα and GM-CSF were added to 5 ng/ml and 100 ng/ml, respectively, to prime the cells. Prior to the ROS assay, an equal volume of prewarmed Detect buffer (DPBS^++++^ containing 16 units/ml horseradish peroxidase (HRP, Sigma, P8375) and 120 μM luminol (Sigma-Aldrich, 123072)) was added to the neutrophils for 3 min at 37 °C. 150 μl/well of the neutrophils/Detect mix were dispensed into a prewarmed 96-well luminometer plate. 100 μl of prewarmed 2.5 × C5a in DPBS^++++^ was added by automatic injection port, and real-time ROS production was recorded at 37 °C. Final assay concentrations were 1.5 × 10^6^ neutrophils/ml and 25 nM C5a. ROS production was quantified by integrating the area under the curve (AUC) of the ROS response over 5 min. IC_50_s were generated using the curve fitting function of GraphPad.

### Data collection and statistical analysis

Sample size was determined using power calculations to yield 80% power, based on results of pilot experiments and on previously published data as referenced. Animals were selected for experiments as described under ‘Mice’ according to genotype, sex, and age. Within these criteria, mice were selected at random by the staff of the Biological Support Unit. Experiments were performed at least three times. Statistical analysis and plotting of graphs were done in GraphPad Prism 10. Data were tested for normality of distribution to determine if parametric or non-parametric methods of analysis were appropriate. Where warranted, data were log-transformed or square-root transformed prior to analysis. Outliers were identified using Tukey’s test and were excluded from the analysis. Otherwise, only samples with known technical errors were excluded from the analysis. For comparison of two groups, paired Student’s *t* test was used. For comparison of multiple groups, two-way ANOVA was used with repeated measures followed by the *post hoc* multiple comparisons correction test recommended by GraphPad. Statistical analysis was done on raw data. Where it was necessary to compare normalized data, the normalized values were excluded from the analysis. Parameters with values of *p* ≤ 0.05 were considered to differ significantly. Results are presented as mean ± standard error of the mean (SEM). In the figures, *p*-values in black denote significant differences, *p*-values in gray are non-significant. Sample size and the numbers of independent experiments are detailed in figure legends.

## Data availability

All data are contained within the article.

## Supporting information

This article contains supporting information.

## Conflict of interest

The authors declare that they have no conflicts of interest with the contents of this article.
