# Supplementary material for: The GPCR adaptor protein Norbin controls the trafficking of C5aR1 and CXCR4 in mouse neutrophils
Source: J Biol Chem. 2024 Oct 28;300(12):107940. doi: 10.1016/j.jbc.2024.107940 (PMC11647504; doi:10.1016/j.jbc.2024.107940)
Supplement: Supporting information [file mmc1.pdf]

Supporting Information

The GPCR adaptor protein Norbin controls the trafficking of C5aR1 and CXCR4 in mouse neutrophils

Stephen A. Chetwynd [1], Richard J. Ward [2], Graeme Milligan [2], Heidi C. E. Welch [1,3]

[1] Signalling Programme, The Babraham Institute, Babraham Research Campus, Cambridge, UK

[2] Centre for Translational Pharmacology, School of Molecular Biosciences, College of Medical, Veterinary and Life Sciences, University of Glasgow, UK

Content:

Supplemental Figures 1 and 2

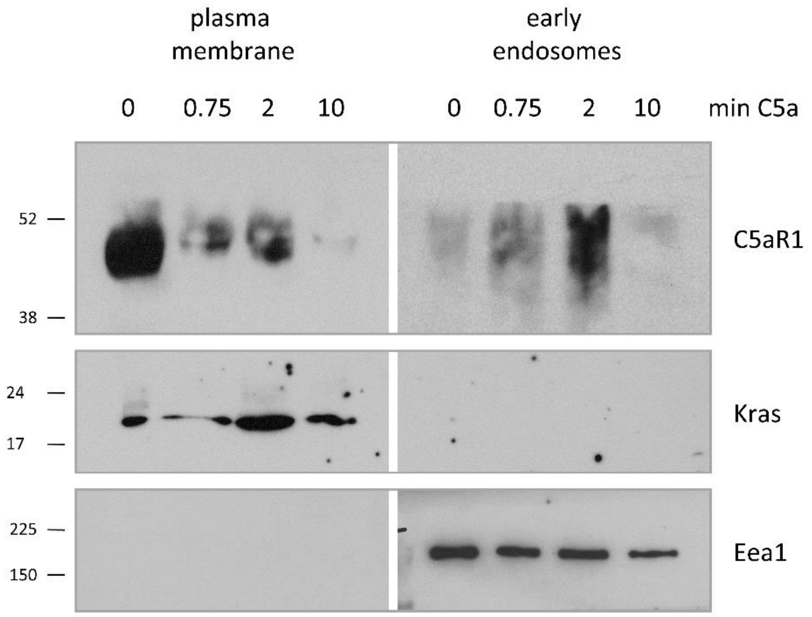

Supplemental Figure 1. C5aR1 translocates from the plasma membrane into early endosomes upon C5a stimulation. Purified *Ncdn*$^{fl/fl}$ neutrophils were stimulated with 15 nM C5a for the indicated periods of time. Early endosomes were isolated from the PGS using Eea1 immunoprecipitation before the plasma membrane was isolated from the endosome-depleted PGS by ultracentrifugation. The same cell-equivalents of plasma membrane and early endosome fractions were loaded. Samples were western blotted with C5aR1, Kras and Eea1 antibodies. Blots shown are representative of 2 independent experiments.

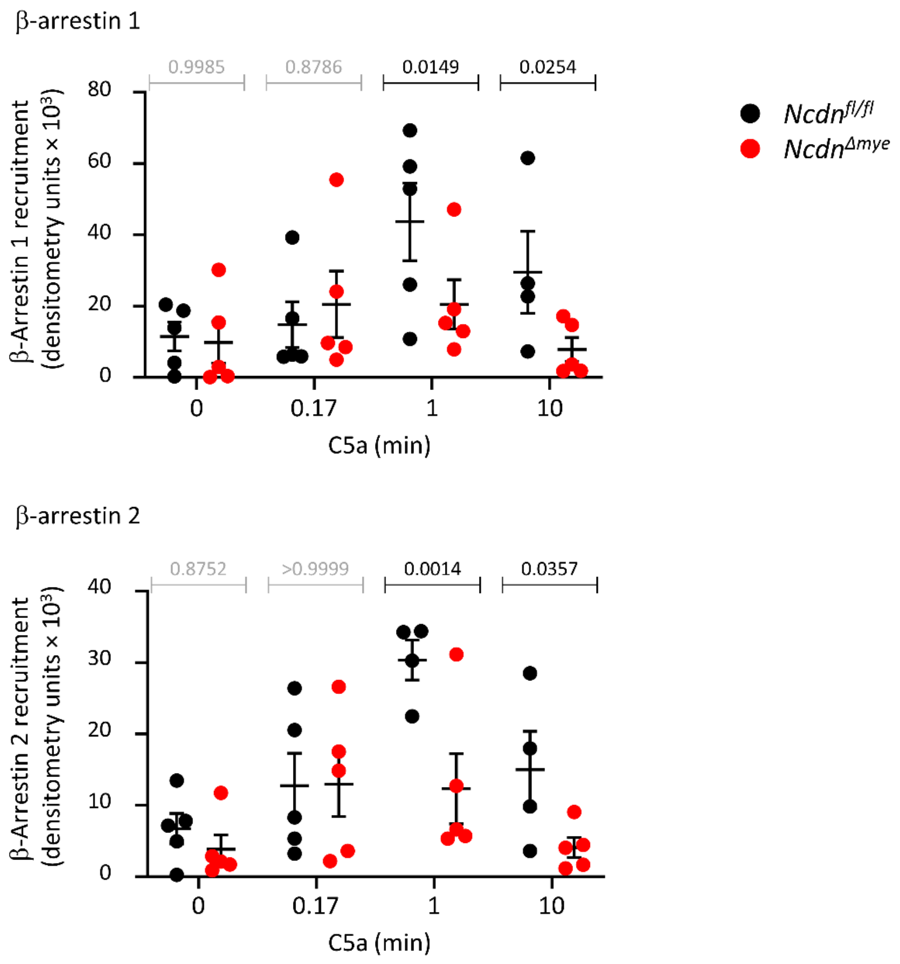

Supplemental Figure 2. Norbin is required for the recruitment of β1- and β2-arrestin. Quantification of data from Figure 6A for β1-arrestin and β2-arrestin recruitment in response to C5a stimulation. Purified *Ncdn*$^{fl/fl}$ (black) and *Ncdn*$^{\Delta mye}$ (red) neutrophils were stimulated with 15 nM C5a for the indicated periods of time, and glycosylated proteins were isolated from the PGS and western blotted with β-arrestin1/2 antibody as described in the legend to Figure 6A. The 47 kDa β1-arrestin and 46 kDa β2-arrestin bands were quantified separately by Fiji densitometry. Data are mean ± SEM of 5 independent experiments; each dot is the mean of 1 experiment. Statistics are two-way ANOVA with Šidák's multiple comparisons test. P-values in black show significant differences, p-values in grey are not significant.
